# Asymmetrically Coordinated Dual‐Atom Manganese Contrast Agents Enabling High‐Efficiency T1 Enhanced MRI for Precise Tumor Visualization

**DOI:** 10.1002/advs.75644

**Published:** 2026-05-29

**Authors:** Ding Yang, Qing‐Yang Li, Jianli Liang, Gengyou Li, Nan Sun, Qianqian Song, Haitao Zhu, Xiaoting Li, Zechuan Li, Jiankai Dong, Weisheng Guo, Yafang Xiao, Chun‐Sing Lee, Ying‐Shi Sun

**Affiliations:** ^1^ Department of Radiology Key Laboratory of Carcinogenesis and Translational Research (Ministry of Education/Beijing) Peking University Cancer Hospital and Institute Beijing P. R. China; ^2^ Center of Super‐Diamond and Advanced Films (COSDAF) & Department of Chemistry City University of Hong Kong Kowloon Hong Kong P. R. China; ^3^ College of Physics and Materials Science Tianjin Normal University Tianjin P. R. China; ^4^ Department of Cardiology Guangzhou Institute of Cardiovascular Disease Guangdong Key Laboratory of Vascular Diseases The Second Affiliated Hospital Guangzhou Medical University Guangzhou P. R. China; ^5^ Department of Minimally Invasive Interventional Radiology The Second Affiliated Hospital School of Biomedical Engineering Guangzhou Medical University Guangzhou P. R. China

**Keywords:** asymmetric coordination geometry, dual‐atom manganese, hepatocyte‐specific imaging, hierarchical boron nitride, MRI contrast agents

## Abstract

Achieving atomically precise control over paramagnetic coordination environments is challenging because relaxivity's sixth power dependence on metal water distance demands sub‐angstrom precision while also requiring rapid water exchange, two inherently competing requirements in conventional systems. Here, leveraging dual‐atom coordination engineering as a rational material design strategy, we successfully synthesized a novel dual‐atom manganese‐based platform featuring a well‐defined asymmetric Mn_2_‐N_3_O_3_ geometry anchored on hierarchical boron nitride (Mn‐BN). Notably, this atomic‐level engineering achieves T1 relaxivity of 36.27 mM^−1^ s^−1^ at 3.0 T and 15.86 mM^−1^ s^−1^ at 7.0 T, approximately 10‐fold higher than those of clinical agents. Combining advanced characterizations with theoretical calculations reveals that adjacent asymmetrically coordinated Mn centers generate cooperative electronic effects that lower water adsorption energy and shorten the Mn–H distance, thereby enhancing water exchange and optimizing dipole–dipole interactions. The Mn‐BN exhibits specific magnetic resonance imaging (MRI) T1 signals for accurate tumor boundary visualization, meanwhile preferential hepatocyte uptake through specific transporters creates differential enhancement for sensitive liver metastases detection. This work positions dual‐atom coordination engineering as an effective materials design strategy for precise enhancement of MRI properties, providing new opportunities for developing advanced contrast agents with tailored biological interactions and extended imaging windows.

## Graphical abstract

A novel dual‐atom manganese MRI contrast agent (Mn‐BN) with a unique asymmetric coordination geometry provided a promising platform for sensitive and specific tumor imaging. In mice model, Mn‐BN nanoparticles precisely functioned as both a primary tumor‐specific enhancer in subcutaneous models and a hepatocyte‐specific agent in liver metastases models to clearly define the tumor delineation on non‐invasive medical imaging.

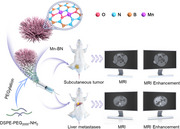

## 1 Introduction

Precise distributions of metal atoms can affect the atomic utilization efficiency, density, and efficiency of exposed active sites and have attracted considerable attention [1, 2, 3, 4]. However, few studies have clearly elucidated how variations in atomic‐level structural precision modulate the underlying mechanisms that govern contrast enhancement and overall imaging performance in magnetic resonance imaging (MRI) [5, 6, 7, 8]. Paramagnetic coordination environments represent a frontier challenge in materials design, where metal center accessibility, electronic structure, and guest molecule interactions must be simultaneously optimized to achieve enhanced functional performance [9]. Atomically precise controlled paramagnetic material is an ideal candidate for contrast‐enhanced MRI, the clinically preferred noninvasive diagnostic technology, particularly because relaxivity—the key performance metric, exhibits a sixth‐power dependence on metal‐water distances and strong sensitivity to coordination geometry [10, 11, 12]. This fundamental relationship makes atomic‐level structural control critical for optimizing contrast agent performance. Current materials design strategies are fundamentally constrained by the difficulty of achieving high metal utilization, optimal coordination geometry, and rapid water exchange at the same time [13, 14]. Overcoming these challenges requires entirely new design concepts rather than incremental improvements to existing systems.

Existing paramagnetic materials operate under water exchange vs. thermodynamic stability trade‐offs that limit performance optimization. Gadolinium chelates achieve structural predictability through rigid coordination cages but suffer from restricted water accessibility and slow exchange kinetics that substantially limit achievable relaxivity [13, 15, 16]. Manganese‐based systems can possess superior electronic properties because their five unpaired 3d electrons provide optimal magnetic moments. However, conventional synthetic approaches using molecular complexes or bulk nanoparticles provide limited control over atomic dispersion and coordination control [17, 18, 19]. These limitations are particularly pronounced in hepatocyte‐targeting applications where both high relaxivity and biological selectivity are required, highlighting the need for materials innovation that may overcome conventional synthetic limitations [20, 21]. The challenge lies in developing synthetic strategies that can precisely control coordination environments while maintaining material stability and biological compatibility.

The emergence of atomically dispersed materials has significantly advanced heterogeneous catalysis by demonstrating how precise control of metal coordination environments enables exceptional activity and selectivity [22, 23, 24]. Single‐atom systems achieve maximum atomic utilization while providing well‐defined active sites for mechanistic understanding and rational optimization. Dual‐atom configurations represent an emerging approach where cooperative electronic effects between proximal metal centers can enhance performance beyond single‐atom limitations through synergistic substrate interactions and enhanced structural stability [25, 26]. However, the application of these materials design principles to paramagnetic systems remains largely unexplored, with only nascent reports on single‐atom contrast agents and limited investigation of coordination effects in MRI materials. This represents a significant opportunity to establish fundamental structure‐performance relationships for atomically dispersed paramagnetic materials.

**FIGURE 1:**
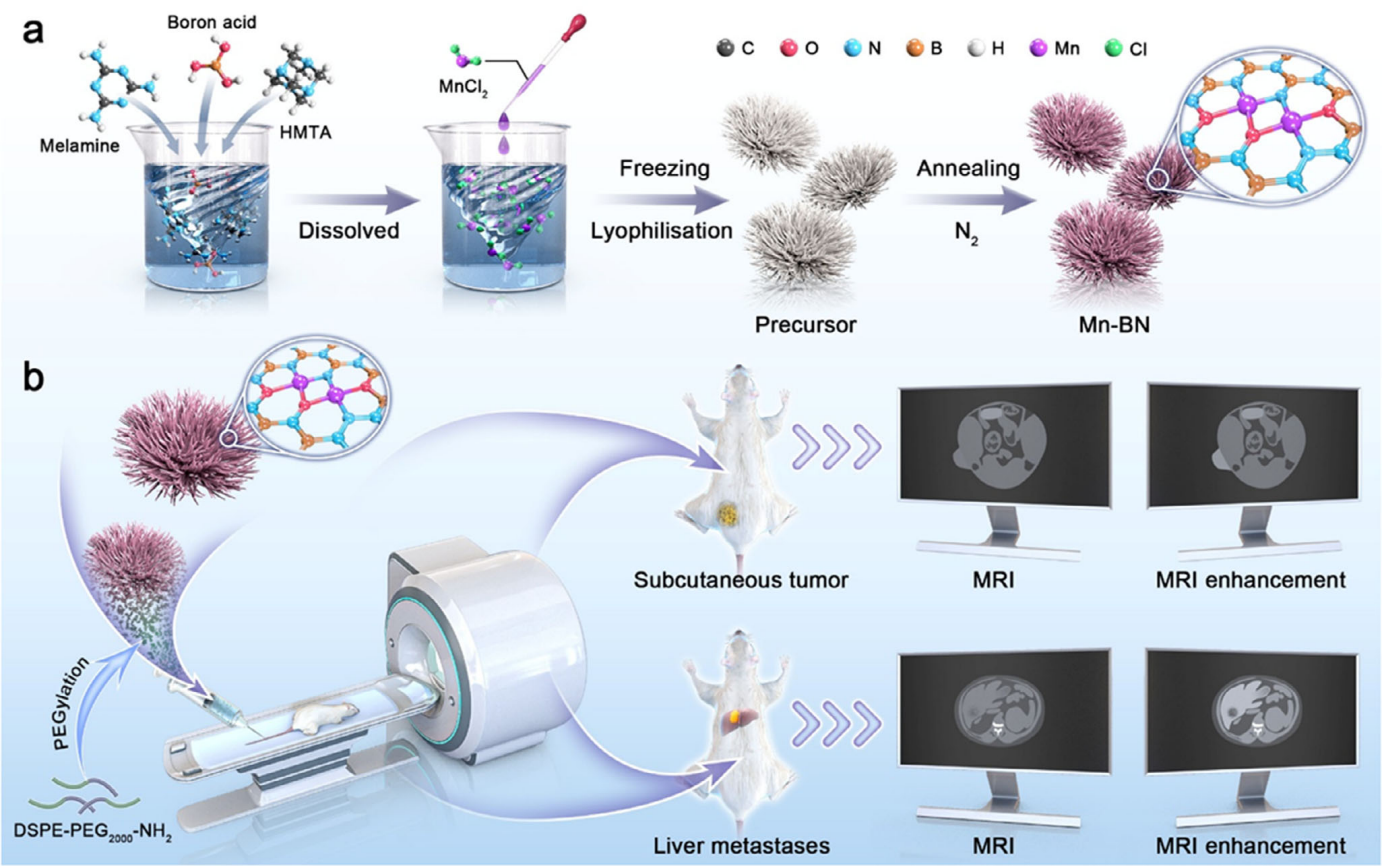
Schematic illustration of Mn‐BN for MRI application. (a) The synthesis process of Mn‐BN; (b) The in vivo validations of Mn‐BN as tumor‐specific and hepatocyte‐specific contrast agents in MRI with subcutaneous tumor and liver metastases models.

Here, we employed dual‐atom coordination engineering as a material design strategy for high‐performance paramagnetic systems, demonstrating controlled synthesis of asymmetric Mn_2_‐N_3_O_3_ configurations on hierarchical boron nitride supports as a novel dual‐atom manganese‐based platform named Mn‐BN (Figure 1a). Through comprehensive structural characterizations, density functional theory (DFT) calculations, and relaxivity measurements, we revealed how dual‐atom geometry directly governs water coordination dynamics and electronic coupling to achieve record‐high relaxivity performance (r1 = 36.27 mM^−1^ s^−1^ at 3.0 T and r1 = 15.86 mM^−1^ s^−1^ at 7.0 T). Moreover, the Mn‐BN performed well in the precise visualization of the tumor boundary. Especially, in liver metastases models, the Mn‐BN exhibited significantly enhanced sensitivity for the detection of metastatic tumors by generating prominent differential enhancement between normal liver tissue and metastatic lesions (Figure 1b). The design principles established in this work, include asymmetric coordination for optimized water exchange, metal‐metal interactions for enhanced stability [27], and hierarchical supports for controlled dispersion, reveal the mechanisms underlying the high relaxivity and provide fundamental guidance for engineering atomically precise paramagnetic materials with tailored properties. By addressing the critical limitations of conventional contrast agents, this materials innovation platform opens new possibilities for earlier detection and more precise characterization of malignancies.

## 2 Results and Discussion

### 2.1 Morphology and Coordination Structure of Dual‐Atom Mn‐BN

**FIGURE 2:**
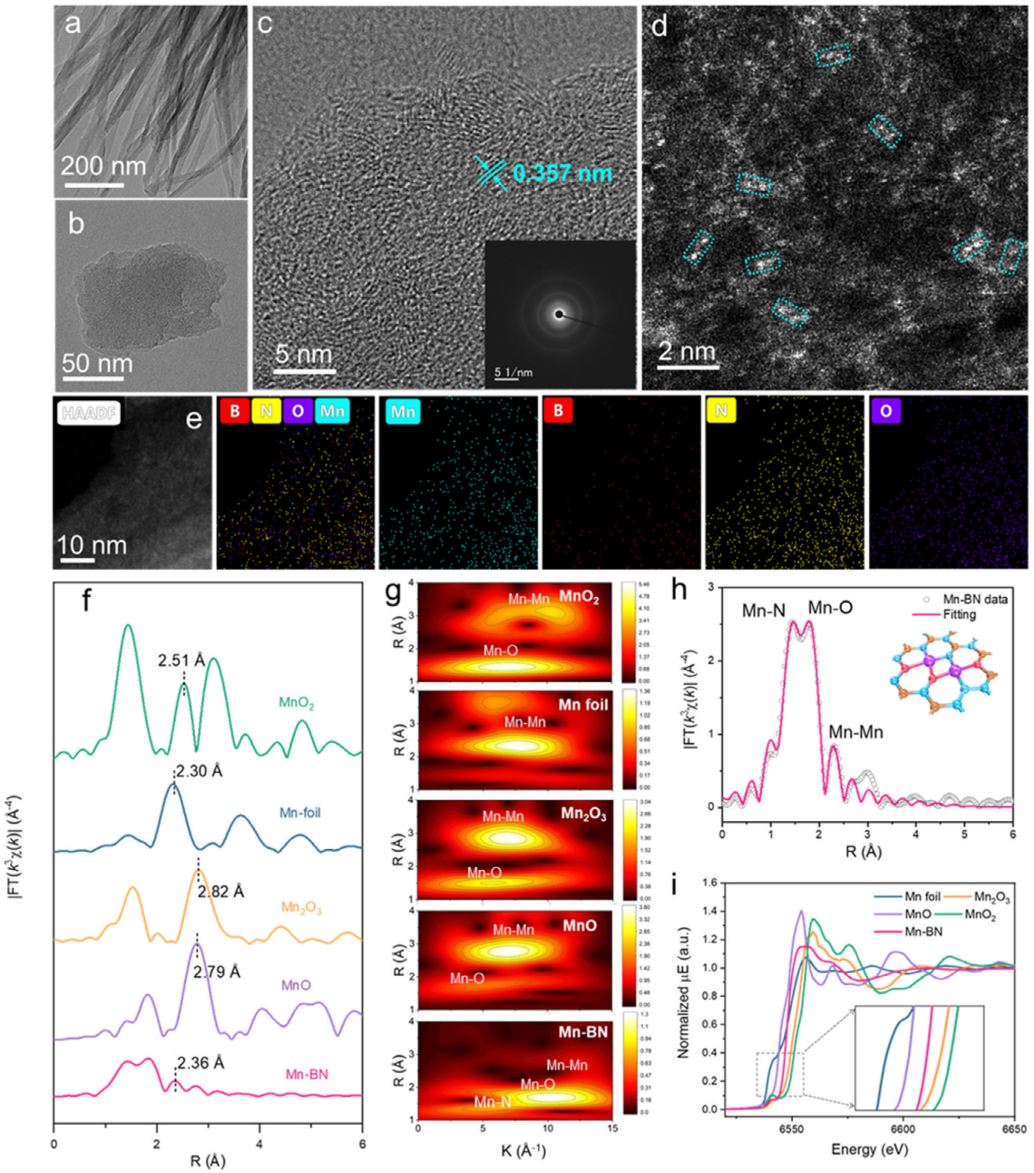
Characteristics of Mn‐BN. TEM image of (a) Mn‐BN and (b) Mn‐BN nanoflakes, (c) HRTEM image and SAED pattern of Mn‐BN (inset); (d) Aberration‐corrected HAADF‐STEM image and (e) corresponding high‐resolution elemental mapping images of Mn‐BN. (f) Fourier transform of the Mn K‐edge EXAFS and (g) Wavelet transform of the K3 weighted EXAFS data of Mn‐BN and reference samples of Mn foil, MnO, Mn_2_O_3_, and MnO_2_. (h) EXAFS fitting curve of Mn‐BN in R space (the inset was the structural model of Mn‐BN). (i) Mn K‐edge XANES.

Details of the material synthesis are provided in the supporting information. Scanning electron microscopy (SEM) and transmission electron microscopy (TEM) were applied to characterize the morphology and structure of the synthesized bulk Mn‐BN material. The SEM and TEM images (Figure 2a; Figure S1a–c) indicate that the bulk Mn‐BN exhibits a flower‐like hierarchical structure comprising highly interconnected nanofibers with a diameter of approximately 50 nm. To ensure the feasibility of subsequent evaluation, the Mn‐BN flower was physically broken down into nanoflakes via ultrasonication (Figure 2b). High‐resolution transmission electron microscopy (HRTEM) (Figure 2c), selected area electron diffraction (SAED) (Inset of Figure 2c), and X‐ray diffraction (XRD) pattern (Figure S2) indicate that the low crystallinity of the as‐prepared Mn‐BN, and there was no Mn metal/Mn oxide detected. Corresponding elemental mapping images of Mn‐BN flower and Mn‐BN nanoflakes (Figures S3 and S4) demonstrate that the Mn element is evenly dispersed throughout the Mn‐BN nanostructure. Aberration‐corrected STEM was used for further investigation of the distribution of the Mn element. In the aberration‐corrected high‐angle annular dark‐field scanning transmission electron microscopy (HAADF‐STEM) image (Figure 2d; Figure S5), a high density of bright spots can be identified, indicating a homogeneous distribution of the Mn atoms throughout the nanostructure, both for Mn‐BN flower and Mn‐BN nanoflakes. Notably, most Mn species appeared as atom pairs (highlighted with blue rectangles in Figure 2g), indicating that dual‐atom Mn sites exist in the sample. Furthermore, the corresponding high‐resolution elemental mapping images (Figure 2e) provide additional confirmation that the uniform distribution of Mn atoms across the boron nitride (BN) nano‐scaffold.

The precise coordination geometry and chemical state of the engineered dual‐atom Mn configuration in Mn‐BN were comprehensively investigated using X‐ray absorption fine spectroscopy (XAFS). The *K^3^*‐weighted Fourier extended X‐ray absorption fine structure (EXAFS) results (Figure 2f) reveal that the main peaks between 1–2 Å, which are characteristic Mn─O/N coordination bonds corresponding to different bond lengths in the Mn‐BN structure [28, 29, 30]. Notably, a small peak at about 2.36 Å was observed in Mn‐BN, which is larger than that of Mn‐Mn in Mn foil. This is likely due to its dual‐atom configuration, in which the two Mn atoms are bridged by ligands rather than directly connected by metallic bond [31]. The wavelet transforms (WT) EXAFS contour plots of Mn‐BN compared with reference materials (Figure 2g) further validate the distinctive coordination environment of the Mn‐BN structure, showing intensity patterns that differ fundamentally from those of Mn foil or bulk Mn oxides. These complementary characterization techniques collectively validate the successful formation of the strategic dual‐atom Mn configuration within the BN framework. The surface compositions and chemical states of Mn‐BN flower and Mn‐BN nanoflakes were investigated by X‐ray photoelectron spectroscopy (XPS). The high‐resolution B 1s, N 1s, and O 1s XPS spectra of Mn‐BN flower show the presence of B‐N (190.8 eV), B‐O (192.3 eV), N‐B (398.3 eV), B‐N‐O (400.4 eV) bonds [32] and N‐Mn (399.4 eV) [33], O‐Mn (530 eV) bonds [34] (Figure S6a–c). The XPS spectra of Mn 2p, B 1s, and N 1s exhibit nearly identical peak positions and shapes of Mn‐BN nanoflakes (Figure S7). Analysis of the local coordination environment of the Mn atom through EXAFS fitting was carried out to obtain definitive evidence for the coordination geometry of Mn atom (Figure 2h; Figures S8 and S9). The data reveal that each Mn center possesses an asymmetrical coordination configuration with average coordination numbers (CN) of Mn─N and Mn─O determined to be 2.1 and 1.8, respectively. Bond lengths were determined to be 1.92 Å for Mn─N and 2.16 Å for Mn─O bonds (Table S1) by fitting the EXAFS spectrum. Critically, the analysis identified Mn‐Mn interactions with an average coordination number of 0.9 and a bond length of 2.39 Å, conclusively establishing the formation of dual‐atom Mn pairs where each Mn atom coordinates preferentially with another Mn atom along with approximately two N atoms and two O atoms, forming the characteristic Mn_2_‐N_3_O_3_ structure (inset of Figure 2h). Chemical state of Mn atoms in Mn‐BN were further studied using XANES, XPS, and ESR characterizations. As shown in Figure 2i, the Mn K‐edge XANES spectrum edge energy for Mn‐BN is situated between MnO and Mn_2_O_3_, implying that the oxidation state of Mn lies between +2 and +3. This aligns with the high‐resolution Mn 2p XPS results, as the specific shakeup satellite peak depicted in Figure S6d is closely linked to Mn^2+^ [35]. Additionally, the identification of a six‐line pattern attributed to isotropic hyperfine splitting in Mn^2+^ can be noted in the electron spin resonance (ESR) spectrum, suggesting that isolated Mn^2+^ have been integrated into Mn‐BN and there are no obvious direct Mn‐Mn metallic bonds (Figure S10) [36].

To enhance the stability and biocompatibility of Mn‐BN for in vivo MRI, we further encapsulated the Mn‐BN nanoflakes with 1,2‐distearoyl‐sn‐glycero‐3‐phosphoethanolamine‐N‐[amino(polyethyleneglycol)‐2000] (DSPE‐PEG_2000_‐NH_2_) to collect the Mn‐BN nanoparticles. The Mn^2+^ releasing rate of Mn‐BN nanoparticles was about 30% in the weakly acidic tumor microenvironment, where the releasing rates were over 60% in conventional Mn‐oxide nanoparticles [37, 38]. The distinctive dual‐atom Mn_2_‐N_3_O_3_ coordination confers enhanced stability to the anchored Mn atoms and ensures minimal Mn^2+^ release under physiological conditions over 72 h, addressing a critical limitation of conventional Mn‐based contrast agents. The hydrodynamic diameter and ζ potential of PEGylated Mn‐BN were respectively determined using dynamic light scattering (DLS) and Zeta potentiometer, to be about 150 nm and ‐6.4 mV, and maintain excellent morphological stability with consistent sub‐200 nm diameter (Figure S11).

### 2.2 Relaxivity Measurement

**FIGURE 3:**
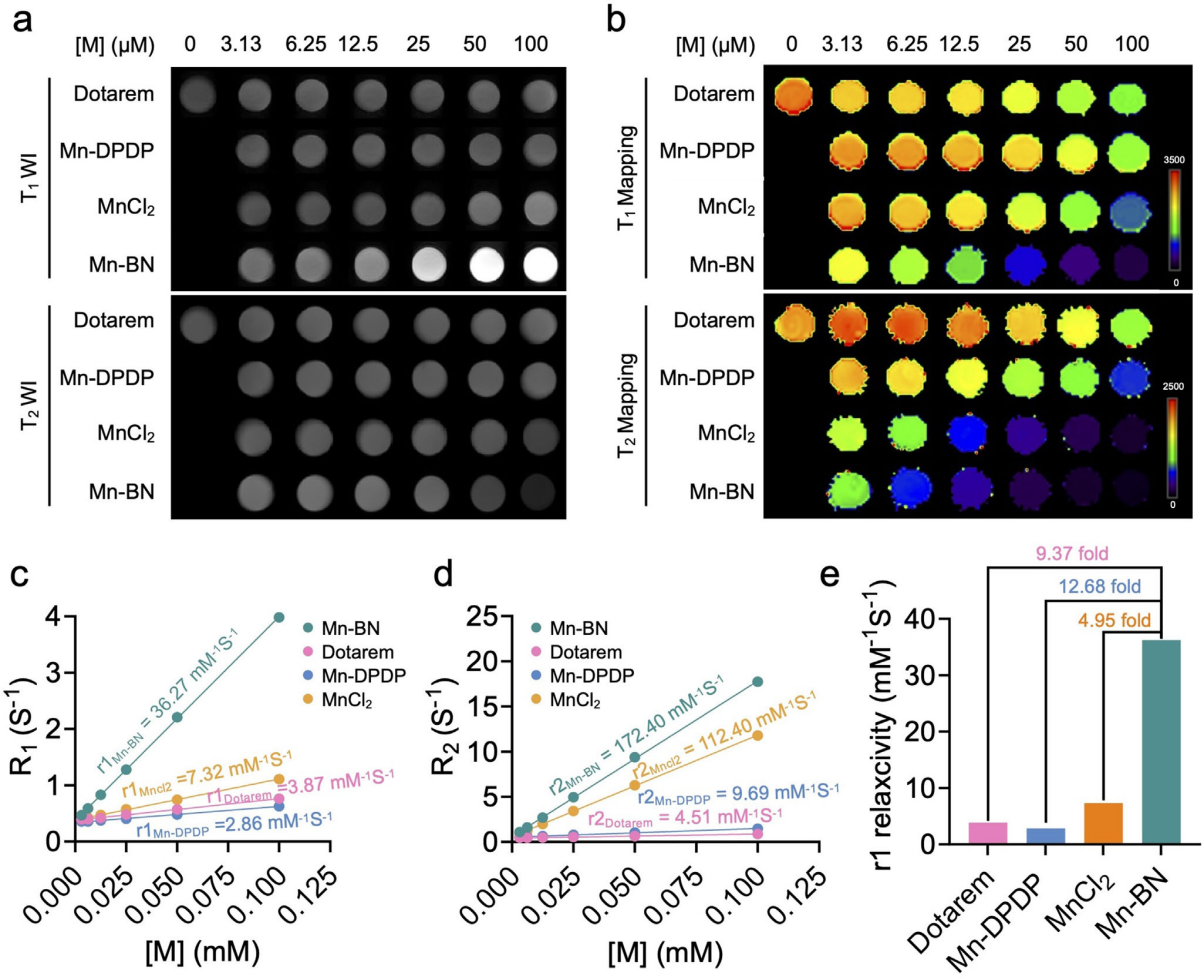
Magnetic properties. (a) T1WI and T2WI phantoms of Mn‐BN, Mn‐DPDP, MnCl_2_, and Dotarem in aqueous solution with different metal concentrations; (b) T1 mapping and T2 mapping phantoms of Mn‐BN, Mn‐DPDP, MnCl_2_, and Dotarem in aqueous solution with different metal concentrations; Metal concentration‐dependent corresponding relaxation time R1(c) and R2(d) at 3T; (e) Comparison of r1 relaxivities at 3T between Mn‐BN with Mn‐DPDP, MnCl_2_, and Dotarem.

The engineered dual‐atom Mn^2+^ with its unique Mn_2_‐N_3_O_3_ coordination geometry in Mn‐BN exhibits exceptional paramagnetic properties stemming from the optimized electronic configuration of five unpaired 3d electrons in each Mn atom center [39, 40]. The Mn‐BN with a distinctive coordination environment was evaluated as an MRI contrast agent using clinical 3T scanner phantom imaging protocols. Concentration‐dependent T1 weighted and T2 weighted phantom imaging reveal that the Mn‐BN functions effectively as a dual contrast agent, with superior enhancement compared to commercial agents such as Dotarem, MnCl_2_, and Mn‐DPDP at 3T (Figure 3a,b).

Relaxivity performances, the fundamental metric for MRI contrast agents, were comprehensively evaluated in both 3T and 7T fields using quantitative T1 mapping and T2 mapping analyses. The results demonstrate that the engineered dual‐atom structure generates unprecedented relaxivity values, with r1 and r2 reaching 36.27 mM^−1^ s^−1^ and 172.40 mM^−1^ s^−1^ at 3T, respectively (Figure 3c,d). These values represent remarkable enhancements, with r1 being 9.37 and 12.68 times higher than clinical Dotarem and Mn‐DPDP agents, respectively, and even 4.95 times higher than MnCl_2_ (Figure 3e). Notably, Mn‐BN also outperformed the referenced MnSA (r1 = 34.14 mM^−1^ s^−1^), where MnSA represents a synthesized single‐atom Mn anchored BN with a Mn─N_4_ coordination environment (Figure S12). Comparison between Mn‐BN and MnSA at 3T was shown in Figure S14a,c, highlighting the superior efficiency of the dual‐atom coordination configuration in modulating proton relaxation, and the relaxivity of these Mn‐based contrast agents was summarized in Table S2. This superior performance aligns with experimental findings, showing that isolated active sites with well‐defined coordination geometry exhibit much higher activity than conventional metal/metal oxide nanoparticles [41]. Beyond the contrast agents mentioned above, the dual‐atom Mn‐BN achieves nearly 10‐fold higher longitudinal relaxivity than FDA‐approved agents and even outperforms the leading single‐atom agent feGd‐N_x_C (r1 = 34.20 mM^−1^ s^−1^ at 3T). Among recently investigated advanced Mn‐based and single‐atom MRI contrast agents, Mn‐BN demonstrates notably superior relaxivity performance (Table S3).

Meanwhile, r2 values of Mn‐BN in an aqueous solution were 38.23 times, 17.79 times, and 1.53 times higher than Dotarem, Mn‐DPDP, and MnCl_2_, respectively (Figure S13). This superior performance illustrates that Mn‐BN is an attractive and promising dual contrast agent candidate for improving clinical MRI diagnosis. Moreover, with the development of MRI scanners, high‐field MRI demands are increased by providing high resolution MRIs for precise diagnosis, but this lacks the support of traditional contrast agents with satisfactory performance [42, 43]. The phantom images of Mn‐BN at high‐field (7T) further showed its high potential as a contrast agent for future clinical application. The superior T1/T2 dual contrast enhancement of the Mn‐BN was also observed at 7T T1 weighted and T2 weighted images compared to the mentioned Mn‐based contrast agents (Figure S14b,d,e). At this high‐field, Mn‐BN maintains the highest relaxivity with r1 and r2 values of 15.86 mM^−1^ s^−1^ and 199.50 mM^−1^ s^−1^, respectively, consistently surpassing MnSA (r1 = 13.47 mM^−1^ s^−1^, r2 = 171.70 mM^−1^ s^−1^), MnCl_2_, and Mn‐DPDP (Figure S14b). The superior relaxivity of Mn‐BN hierarchy highlights the transformative potential of atomically dispersed metal engineering for next‐generation MRI clinical contrast agents for increasing the contrast between tissues.

### 2.3 Biocompatibility of Dual‐Atom Mn‐BN

The suitability of Mn‐BN for biomedical applications at diagnostic concentrations was confirmed through complementary in vitro (Figures S15 and S16) and in vivo biocompatibility assessments. After direct injection, no obvious acute toxicity incidence, and negligible damage is shown in hematology analysis, blood biochemistry, and main organs hematoxylin and eosin (H&E) staining results after 7 days (Figures S17 and S18). However, the mice in the MnCl_2_ group shows significant abnormality of blood indexes at the same dose induced. The Mn‐BN illustrates its superior biosafe capability as the Mn^2+^ delivery system to mitigate its breaking of the blood‐brain barrier in the analysis of brain tissues. In the MnCl_2_ group, the observed cellular stress was in the neuronal populations and a potential neurotoxin effect with free Mn^2+^ exposure. Moreover, we evaluated the Mn accumulation in the brain by the in vivo MRI tumor imaging, which shows a notable enhancement after 4 h of MnCl_2_ injection, but which does not occur in the Mn‐BN group (Figure S19). The long‐term biocompatibility of Mn‐BN further confirms its high potential to improve diagnostic efficacy in future clinical biomedical imaging applications.

### 2.4 Tumor‐Specific Contrast Agents

**FIGURE 4:**
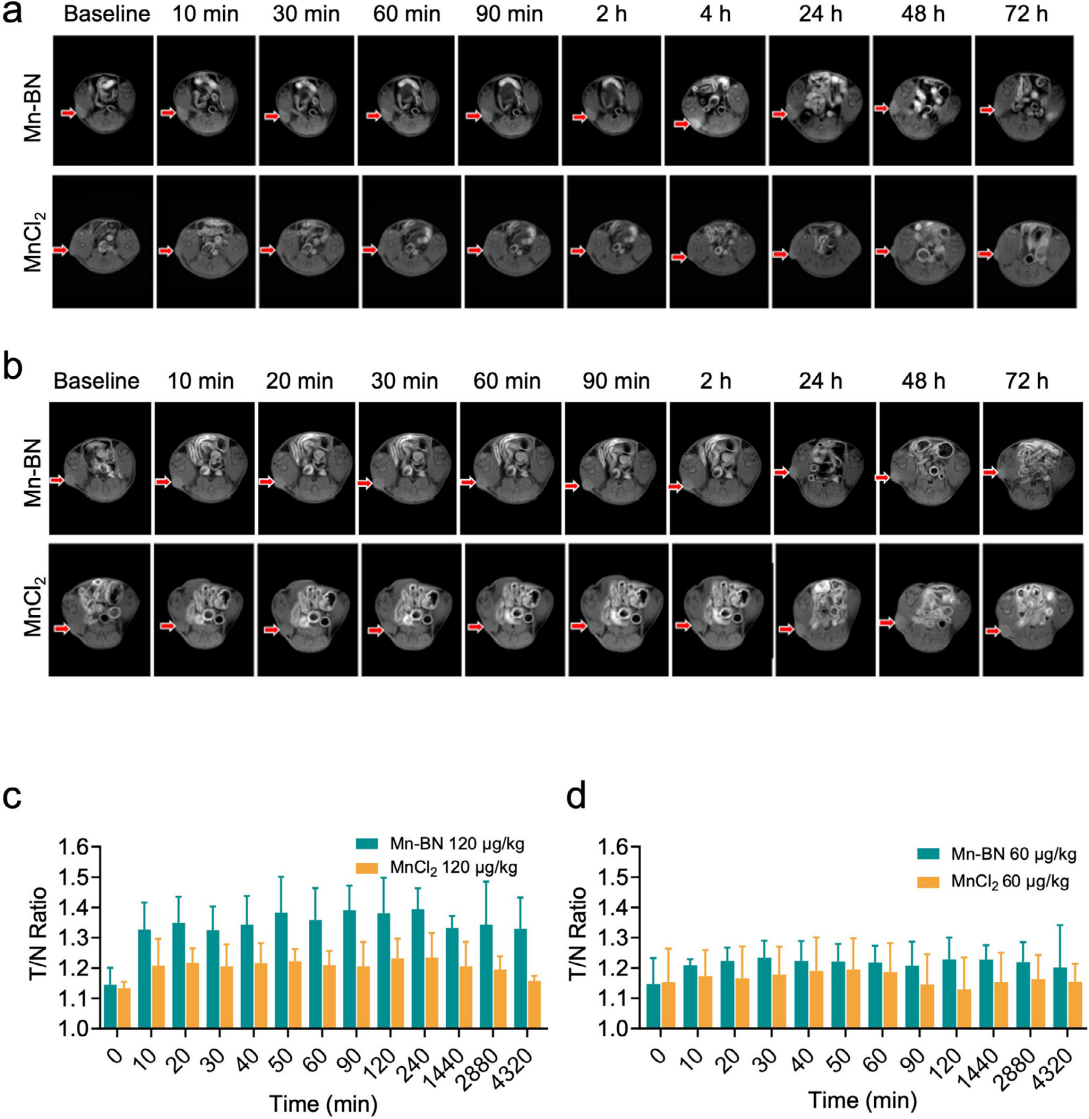
Contrast‐enhanced MRI of BALB/c mice bearing subcutaneous tumor (4T1 cells) with Mn‐BN and MnCl_2_ at 3T. Axial T1 weighted MR images acquired at different time points after intravenous injection of Mn‐BN or MnCl_2_ in the dose group of (a) 120 µg/kg Mn per mouse, and (b) 60 µg/kg Mn per mouse; Corresponding T/N contrast ratios of T1 signal in dose group of (c) 120 µg/kg Mn per mouse dose group, and (d) 60 µg/kg Mn per mouse dose group (n = 3 for each group).

To demonstrate the Mn‐BN enhancement of MRI in vivo, the dual‐atom Mn‐BN was physically dispersed and PEGylated with DSPE‐PEG_2000_‐NH_2_ to optimize biocompatibility and circulation properties. We first evaluated the enhancement in the mouse primary tumor model with tail intravenous injection (i. v.) of the contrast agents. The MnCl_2_ was introduced in the control group considering its mentioned higher enhancement performance and more intrinsically active paramagnetic centers. Female BALB/c mice bearing 4T1 subcutaneous tumor with a tumor size of around 100 mm^3^ were introduced for tumor‐specific enhancement MRI scanning. The coronal T1 weighted MR images were obtained in the middle dose (120 µg/kg Mn) and high dose (240 µg/kg Mn) groups for 24 h to track the nano contrast enhancement signal in abdominal MRI between organs, and MnCl_2_ (120 µg/kg Mn), administrated in the control group. Higher signal amplification is observed in the liver, kidney, gall bladder, and tumor (Figure S20) compared with the MnCl_2_ group, while the Mn contrast enhancement is retained in the tumor after 24 h of injection, but there is no detectable retention contrast T1 signal intensity enhancement in the liver and kidney, and the blood circulation half‐life is about 45 min (Figure S21). The extremely high T1 intensity enhancement is shown in the gall bladder, which may be triggered by the primary excretion of excess Mn via the biliary excretion route [44]. For further investigation of tumor‐specific contrast enhancement, T1 weighted axial images were obtained with tumor models before and after contrast injection. In the middle dose group, a clear boundary between tumor and normal tissue, especially with the near muscle tissue, has been defined with the Mn‐BN contrast enhancement images, which peak enhancement after 4 h of Mn‐BN injection (Figure 4a). Then, the tumor‐to‐normal tissue contrast ratio (T/N) was adopted to illustrate the enhancement intuitively; the ratio reached over 1.3 posts 10 min of injection and peaked at 4 h post‐injection. The high ratio of Mn‐BN was kept for 3 days (Figure 4c) possibly due to the leaky vasculature and impaired lymphatic drainage in tumor tissue [45]. Also, the enhancement of tumor tissue was observed in the low dose group (i.v., 60 µg/kg Mn per mouse) of Mn‐BN (Figure 4b,d). For both groups, tumor enhancement of Mn‐BN is prominent than that of MnCl_2_ with a more sensitive, brighter signal and longer imaging window duration. Additionally, in 24 h post administration of Mn‐BN, the tumor enhancement is positive dose‐dependent on the agents (Figure S22). The significant enhancement in tumor tissues with shown bright boundary illustrates it as a potential MRI contrast agent to support clinically precise diagnosis for the primary tumors.

### 2.5 Hepatocyte‐Specific Tumor Contrast Agents

Beyond the primary tumor model, the detection of metastatic lesions presents a substantially greater clinical challenge [46], particularly in the liver, which indicates a poor prognosis. These metastases predominantly manifest as multiple discrete focal lesions, with small lesions particularly challenging to detect at early stages. The late identification directly impacts therapeutic outcomes and patient survival. Clinical evidence consistently demonstrates that detecting early‐stage liver metastases dramatically improves prognosis, yet conventional imaging approaches frequently fail to identify these critical small lesions [47]. This persistent diagnostic gap underscores the urgent need for hepatocyte‐specific contrast agents with enhanced sensitivity for detecting subtle differences between tumor and surrounding normal hepatic parenchyma [48].

The targeting ability of Mn‐BN in bioimaging was investigated in tumor cells and liver cells. The variety of internalization of Mn‐BN nano contrast agents in different cells enhanced the contrast between tissues. After incubation with the murine 4T1 breast adenocarcinoma, and Alpha Mouse Liver 12 (AML‐12) cell lines, respectively, Mn‐BN was gradually internalized by cells. Compared with 4T1 cells, AML‐12 cells exhibited an earlier peak in internalization and a more complete internalization process when co‐cultured with Mn‐BN (Figure S23). In clinical settings, hepatocyte‐specific contrast agents are introduced to specialized MRI as selectively taken up by functioning hepatocytes through specific transporters, thereby enhancing the contrast‐to‐noise ratio and improving the conspicuity of non‐hepatocellular lesions. These results indicate that Mn‐BN is easily taken up by liver cells with expected potential as hepatocyte‐specific contrast agents.

**FIGURE 5:**
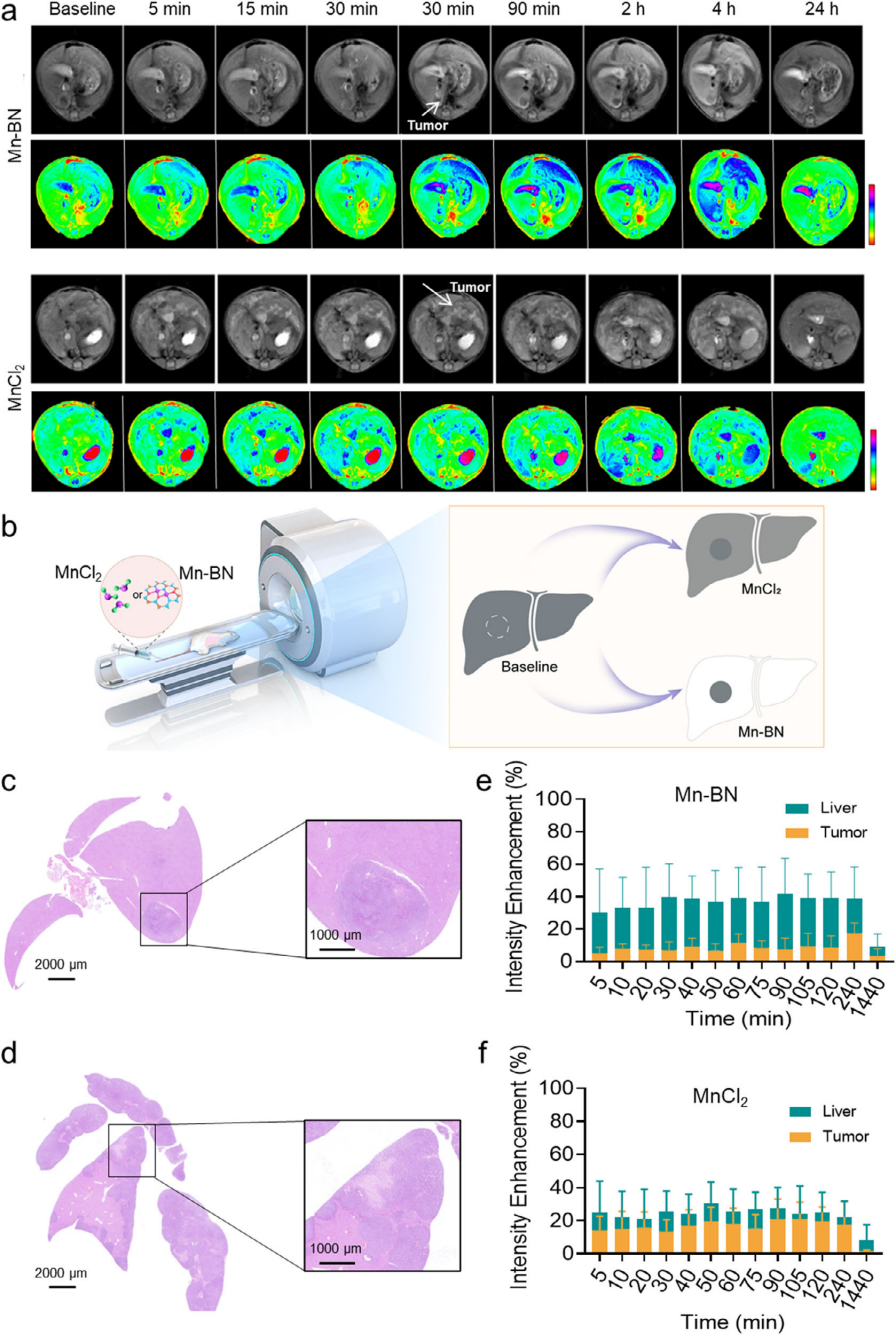
Contrast‐enhanced MRI of BALB/c mice bearing liver metastases (CT26‐luc cells) with Mn‐BN and MnCl_2_ at 3T. Liver metastases model using female BALB/c mice bearing CT26‐Luc hepatic tumors, verified through bioluminescence imaging (PerkinElmer, IVIS Lumina III). (a) Axial T1‐weighted MR images acquired at different time points after intravenous injection of Mn‐BN or MnCl_2_ (120 µg/kg Mn per mouse), T1‐weighted axial upper abdominal images were acquired using a clinical 3T MRI scanner (United Imaging, UMR790) with the mouse coil.; (b) Schematic illustration of comparison between the hepatocyte‐specific contrast enhancement of Mn‐BN and non‐specific enhancement of MnCl_2_ for liver with metastatic tumor tissue; H&E staining for corresponding to MRI of liver with tumor for (c) Mn‐BN and (d) MnCl_2_; Corresponding T1 signal intensity enhancement of liver and tumor tissue with (e) Mn‐BN and (f) MnCl_2_.

The distinctive cellular tropism of Mn‐BN toward functional hepatocytes provides the mechanistic foundation for its application as a hepatocyte‐specific contrast agent. This property creates an ideal scenario for liver metastases detection, where the differential enhancement between hepatic parenchyma and metastatic lesions generates pronounced diagnostic contrast. To evaluate its potential in clinically relevant settings, we established a liver metastases model using female BALB/c mice bearing CT26‐Luc hepatic tumors. Based on preliminary dosage optimization, we administered Mn‐BN intravenously at 120 µg/kg Mn per mouse, with MnCl_2_ at equivalent manganese concentration serving as a control. In the meantime, Mn‐BN‐Cy5 was adopted to observe the biodistribution with in vivo fluorescence imaging to certify the low burden for organs after administering Mn‐BN (Figures S24 and S25). T1‐weighted axial upper abdominal images were acquired, documenting contrast enhancement of the liver over 24 h post‐injection. The Mn‐BN contrast agent demonstrates remarkable hepatocyte‐specific behavior within minutes of administration. Axial T1‐weighted MR images reveal pronounced enhancement of normal hepatic parenchyma (Figure 5a) with signal intensity progressively increasing to maximum levels at 4 h post administration. This differential enhancement creates exceptionally clear boundaries between metastatic lesions and surrounding healthy liver tissue. While the MnCl_2_ control group also shows enhancement, it lacks the critical specificity required for unambiguous tumor delineation, with lesions remaining poorly defined within the enhanced liver background. This superior diagnostic performance is illustrated in a schematic diagram as Figure 5b, highlighting the preferential hepatocyte uptake of the Mn‐BN. Histopathological validation (Figure 5c,d) confirmed excellent correlation between MRI findings and actual tumor distribution, validating the high diagnostic precision of Mn‐BN contrast enhancement. Quantitative analysis of signal intensity demonstrates that in the Mn‐BN group (Figure 5e), normal liver tissue enhancement rapidly reached 30% above baseline, while tumor tissue showed minimal enhancement (<5%). Although a slight additional enhancement is observed in both tissues over the following 4 h, tumor enhancement remained consistently and significantly lower than liver tissue, with peak tumor enhancement never exceeding 20%, creating an ideal contrast differential for sensitive lesion detection. The control MnCl_2_ group (Figure 5f) exhibits fundamentally different enhancement kinetics, with immediate signal intensity increases in both liver (>20%) and tumor tissues (>10%), resulting in poor tissue differentiation. By comparison, the dual‐atom Mn‐BN not only produces significantly higher absolute enhancement of normal liver tissue, but most critically maintains a superior contrast differential between tumor and normal tissue throughout the imaging period. This enhanced contrast specificity can be attributed to the accumulation and sequestration of nano‐sized Mn‐BN in the liver [49]. The difference in distribution for Mn‐BN nanoparticles in metastatic lesions and surrounding healthy liver tissue has been further validated in histological and fluorescence imaging in Figure S26. A particularly noteworthy feature of the Mn‐BN platform is its extended imaging time window, maintaining high‐quality T1 contrast for over 4 h post‐administration. This prolonged visualization capability represents a significant clinical advantage over conventional contrast agents, potentially enabling extended procedural time for image‐guided interventions, surgical planning, and intraoperative navigation. Furthermore, this extended imaging time window offers the possibility of evaluating immediate therapeutic responses through sequential imaging during a single contrast administration. We further applied Mn‐BN to a CCl_4_‐induced liver fibrosis model to test whether its hepatocyte‐selective uptake could resolve diffuse parenchymal disease. The high selectivity and sensitivity of the Mn‐BN were validated by both cellular level (Figure S27) and in vivo MR imaging (Figure S28). The rapid signal enhancement observed in functional hepatic parenchyma within 1 h post‐injection, whereas fibrotic regions remained hypointense, appearing as diffuse areas of lower signal. This contrast between tissues persisted for over 2 h. MnCl_2_, by comparison, enhanced the liver uniformly and failed to distinguish fibrotic from normal tissue. Taken together with the metastases imaging data, these results confirm that the hepatocyte‐selective accumulation of Mn‐BN translates into diagnostic contrast across both focal and diffuse liver pathologies.

The liver metastases and fibrosis models represent two fundamentally different imaging challenges — one requiring detection of discrete focal lesions against an enhanced background, the other demanding visualization of diffuse parenchymal damage. That Mn‐BN performs well in both settings reflects the robustness of its underlying mechanism: selective accumulation in functional hepatocytes, with minimal uptake in pathological tissue regardless of lesion morphology. In the metastases model, the contrast differential between normal liver and tumor tissue was sufficient to resolve lesion boundaries that remained ambiguous with MnCl_2_. In the fibrosis model, the same mechanism rendered fibrotic regions conspicuously hypointense against enhanced functional parenchyma, offering a potential non‐invasive alternative to biopsy for staging fibrotic progression.

Overall, this strategically designed contrast agent demonstrates remarkable versatility in vivo, functioning as both a primary tumor‐specific enhancer in subcutaneous models and a hepatocyte‐specific agent in liver metastases models. In primary subcutaneous tumors, Mn‐BN selectively enhances tumor tissue, creating a clear boundary definition between malignant and normal tissues. In the more challenging liver metastases setting, Mn‐BN demonstrates a combination of advantageous properties, including selective hepatocyte enhancement, superior tumor‐to‐liver contrast, and extended imaging time window. These features establish dual‐atom Mn‐BN as a transformative contrast platform with significant potential to improve the precision of liver metastases diagnosis and therapeutic intervention, ultimately enhancing patient outcomes.

### 2.6 Theoretical Investigations

**FIGURE 6:**
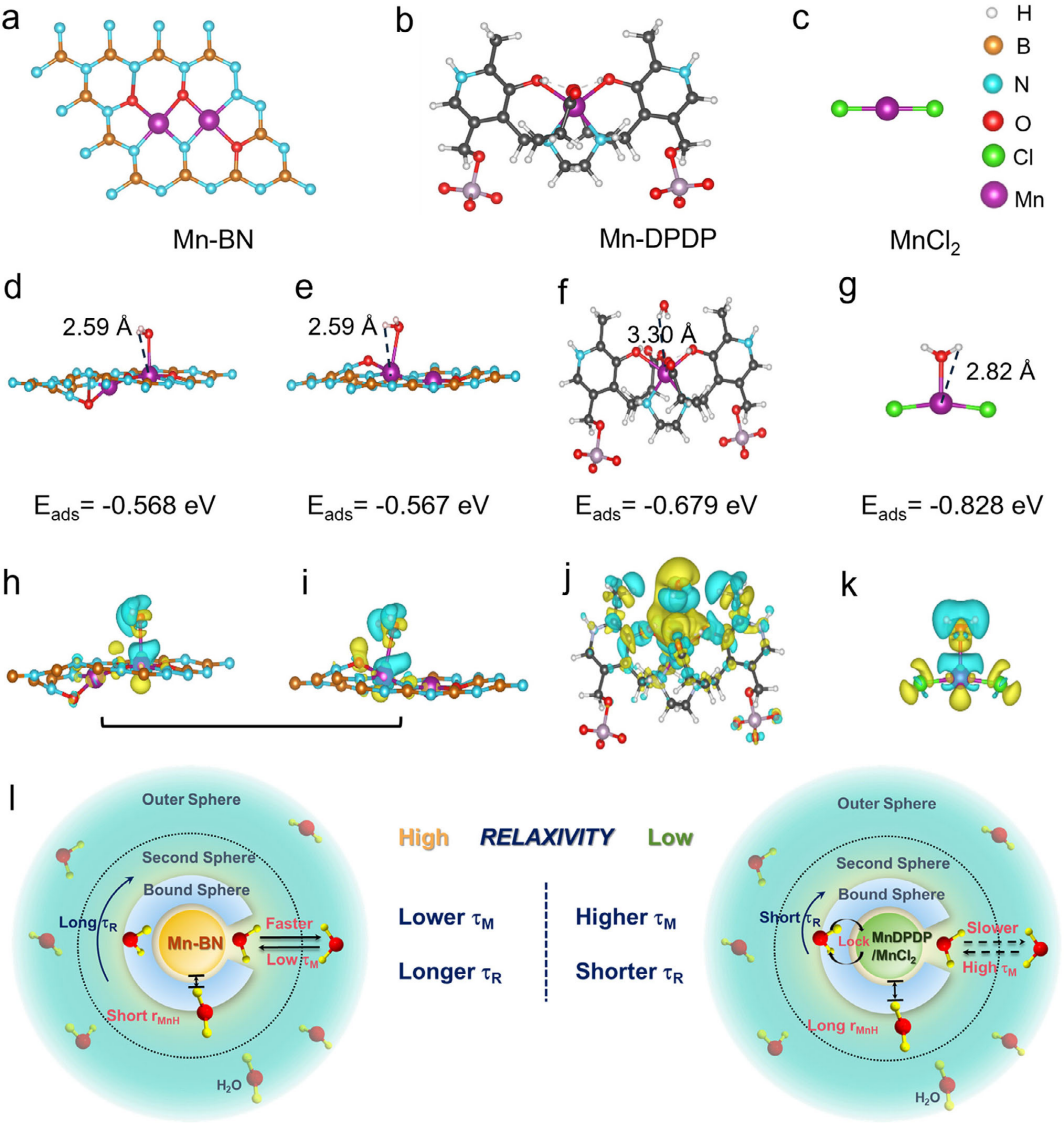
Theoretical calculations. Models for (a) Mn‐BN, (b) Mn‐DPDP and (c) MnCl_2_; The optimized geometry for H_2_O adsorption of (d, e) Mn‐BN, (f) Mn‐DPDP and (g) MnCl_2_ and (h‐k) corresponding electron density difference, where the yellow part represents electron accumulation and the blue part represents electron depletion. (l) A graphical representation of the main factors influencing the r1 relaxivity of Mn‐BN contrast agent.

Dipolar interactions between water proton nuclei and unpaired electron spins cause a strong and fluctuating local magnetic field of Mn, which is primarily accountable for the paramagnetic relaxation of the contrast agents. To further investigate the mechanistic origin of the superior relaxivity enhancement performance of Mn‐BN, DFT calculations were performed using models of MnCl_2_, Mn‐DPDP, and Mn‐BN (Figure 6a–c). The water‐metal direct bound relaxes and transmits the relaxation effect to the bulk water through exchange with another water molecule, which could be governed by an intrinsic factor—the mean residence time of water ligand (τ_M_) [12]. Faster water exchange dynamics between the bound water and the bulk water facilitate more efficient spreading to the surrounding water. Consequently, the relaxivity efficiency of paramagnetic contrast agents could be promoted by accelerating water exchange kinetics and strengthening direct dipolar interaction. The adsorption structures and relative adsorption energy (E_ads_) of H_2_O molecules on Mn‐BN, Mn‐DPDP and MnCl_2_ were shown in Figure 6d–g. It is found that the E_ads_ for H_2_O adsorption on dual‐atom Mn atoms in Mn‐BN are −0.568 and −0.567 eV, respectively, both lower than that of Mn‐DPDP (‐0.679 eV) and MnCl_2_ (−0.828 eV), indicating that the water exchange on Mn‐BN is easier and faster than on the others, leading to the enhancement of the relaxivity. The distance between the Mn atoms to water hydrogen distance (r_MnH_) in Mn‐BN are 2.59 Å, lower than that of Mn‐DPDP (3.30 Å) and MnCl_2_ (2.82 Å). The shortest distance provides the most powerful dipole‐dipole interactions for dramatic enhancement of the relaxivity rate. According to the Solomon‐Bloembergen‐Morgan equations, the relaxation rate is inversely proportional to the sixth power of distance (1/r_MnH_^6^) [12]. Furthermore, the charge density differences of the H_2_O molecule on Mn‐BN, Mn‐DPDP, and MnCl_2_ were calculated to reveal the interaction between H_2_O and the Mn atom. Mn‐BN exhibits localized charge accumulation specifically between the Mn and O atoms (Figure 6h,i), while Mn‐DPDP displays delocalized electron redistribution around adsorbed H_2_O (Figure 6j), indicative of multidirectional ligand interactions that stabilize hydration complexes, thereby impeding rapid water exchange. For MnCl_2_ (Figure 6k), although there is only one interaction between the O atoms of H_2_O and the Mn atoms, the interaction strength between H_2_O and Mn atoms is stronger than Mn‐BN, restricting the water exchange rate. Therefore, the Mn‐BN with the optimal direct and transmit interaction between Mn and H_2_O, performs an excellent T1 relaxivity enhancement property.

In addition, rotational correlation time (τ_R_) is also a key factor influencing T1 relaxivity [50]. An effective strategy to enhance relaxivity is to slow down the rotational motion of contrast agent (increasing τ_R_). This is typically achieved by increasing the volume and mass of contrast agents. Compared with MnCl_2_ and Mn‐DPDP, Mn‐BN nanoparticles have much larger volume and mass; further, Mn ions are immobilized on or within the BN supports via coordinate bonds. These features result in a substantially longer τ_R_ than that of MnCl_2_ and Mn‐DPDP, which is an attribute that directly favors T1 relaxivity enhancement. Collectively, Mn‐BN exhibits lower τ_M_ and longer τ_R_ than those of MnCl_2_ and Mn‐DPDP. These differences in τ_M_ and τ_R_ are the primary reason for the enhanced T1 relaxivity of Mn‐BN, which has been schematically illustrated in Figure 6l.

## 3 Conclusion

In summary, this work has successfully engineered a novel dual‐atom manganese platform Mn‐BN, featuring a distinctive asymmetric coordination geometry structure of dual‐atom Mn_2_‐N_3_O_3_ anchored on hierarchical porous boron nitride nanostructures. The Mn‐BN achieved remarkable relaxivity values (r1 = 36.27 mM^−1^ s^−1^ and 15.86 mM^−1^ s^−1^ at 3.0 T and 7.0 T, respectively) that substantially outperform current clinical commercial contrast agents and most of the reported contrast agents. In comprehensive in vivo evaluation, the Mn‐BN platform demonstrates exceptional versatility, enabling precise visualization of primary tumor boundaries while providing hepatocyte‐specific enhancement for sensitive detection of liver metastases, addressing the critical clinical need for early detection of hepatic metastases. Furthermore, the Mn‐BN provides an extended imaging time window, offering unprecedented opportunities for longitudinal visualization and potential therapeutic monitoring. Comprehensive structural characterizations combined with DFT calculations reveal that the hierarchical BN support ensures the uniform dispersion of Mn atoms, maximizing the utilization of Mn atoms; the asymmetric coordination geometry structure of dual‐atom Mn_2_‐N_3_O_3_ can effectively accelerate water exchange rate, enhance dipole‐dipole interactions, and slow down the rotational motion, thereby achieving higher relaxivity. This work successfully exploits the materials design approach in achieving both fundamental performance breakthroughs and advanced biological functionality, highlighting the potential for atomically precise engineering to overcome traditional limitations across diverse applications. The established principles of controlled dual‐atom coordination, systematic structure‐property optimization, and hierarchical materials integration provide a framework for developing next‐generation functional materials with atomic‐level precision, and multi‐modal performance in complex applications.

## 4 Experimental Method

The detailed experimental method is available in Supporting Information.

## Conflicts of Interest

The authors declare no conflicts of interest.

## Supporting information




**Supporting File**: advs75644‐sup‐0001‐SuppMat.pdf.

## Data Availability

The data that supports the findings of this study are available in the supplementary material of this article.
