# Supplementary material for: Asymmetrically Coordinated Dual‐Atom Manganese Contrast Agents Enabling High‐Efficiency T1 Enhanced MRI for Precise Tumor Visualization
Source: Adv Sci (Weinh). 2026 May 29;13(43):e75644. doi: 10.1002/advs.75644 (PMC13335771; doi:10.1002/advs.75644)
Supplement: Supplementary file 1 — Supporting File: advs75644‐sup‐0001‐SuppMat.pdf. [file ADVS-13-e75644-s001.pdf]

Supporting Information

Asymmetrically Coordinated Dual-Atom Manganese Contrast Agents Enabling High-Efficiency T1 Enhanced MRI for Precise Tumor Visualization

Ding Yang†, Qing-Yang Li†, Jianli Liang†, Gengyou Li, Nan Sun, Qianqian Song, Haitao Zhu, Xiaoting Li, Zechuan Li, Jiankai Dong, Weisheng Guo, Yafang Xiao*, Chun-Sing Lee*, and Ying-Shi Sun**

D. Yang, Dr. Q. Y. Li, N. Sun, Dr. H. T. Zhu, X. T. Li, Z. C. Li, Prof. Y. S. Sun
Department of Radiology, Key Laboratory of Carcinogenesis and Translational Research (Ministry of Education/Beijing), Peking University Cancer Hospital and Institute, No. 52 Fu Cheng Rd, Hai Dian District, Beijing 100142, P. R. China. Email: sys27@163.com.

Dr. J. L. Liang, Prof. C. S. Lee
Center of Super-Diamond and Advanced Films (COSDAF) & Department of Chemistry, City University of Hong Kong, 83 Tat Chee Avenue, Kowloon, 999077, Hong Kong, P. R. China. Email: apcslee@cityu.edu.hk; jliang58-c@my.cityu.edu.hk.

Dr. Q. Q. Song
College of Physics and Materials Science, Tianjin Normal University, Tianjin 300387, P. R. China

G.Y. Li, Dr. J. K. Dong, Prof. Y. F. Xiao
Department of Cardiology, Guangzhou Institute of Cardiovascular Disease, Guangdong Key Laboratory of Vascular Diseases, The Second Affiliated Hospital, Guangzhou Medical University, Guangzhou, 510260, P. R. China. Email: yafangxiao@gzhmu.edu.cn.

Prof. W. S. Guo, Prof. Y. F. Xiao
Department of Minimally Invasive Interventional Radiology, The Second Affiliated Hospital, School of Biomedical Engineering Guangzhou Medical University, Guangzhou 510260, P. R. China.

Experimental details

Synthesis of Mn-BN. The chemicals, consisting of 0.126 g of melamine, 0.124 g of boric acid, and 0.7 g of methenamine (all chemicals were purchased from Sigma Aldrich) were completely dissolved in 45 mL of deionized water within a glass beaker placed in an 80 ºC water bath under continuous stirring. Subsequently, a solution of 5 mL of $MnCl_2·4H_2O$ (8.93 mg, Sigma Aldrich) was gradually introduced into the mixture while stirring. The beaker was then extracted from the water bath and stirred at room temperature (25ºC) for 10 minutes. Following this, the solution in the glass beaker was transferred to a polymer beaker and subsequently frozen with liquid nitrogen before undergoing freeze-drying to acquire Mn-BN precursors. After that, the precursor was placed into a quartz tube in a horizonal tube furnace. The tube underwent evacuation with a mechanical pump until reaching a vacuum of $\sim 10^{-3}$ Torr. High purity N_2 was then introduced into the tube at a flow rate of 100 mL min^{-1} while the mechanical pump operated continuously. The tube was then annealed at 900°C for 4 hours under N_2 flow and pumping. Finally, the Mn-BN was obtained after the tube was naturally cooling to room temperature.

Synthesis of MnSA. The Mn-SA was synthesized through a two-step process involving the preparation of boron nitride (BN) followed by the deposition of manganese (Mn) atoms. First, BN was prepared using the same method as Mn-BN without the addition of Mn salts. Subsequently, 100 mg of the as-prepared BN was dispersed in 50 mL of deionized water under vigorous stirring. A solution containing 10.8 mg of $MnCl_2·4H_2O$ was then slowly added dropwise to the BN dispersion, and the mixture was stirred continuously for 1 hour to ensure homogeneous adsorption of Mn ions onto the BN surface. The resulting solid was collected by centrifugation at high speed and freeze-dried to obtain a fine powder. Finally, the powder was transferred to a tube furnace and annealed at

400°C for 1 hour under a nitrogen atmosphere. After natural cooling to room temperature, the MnSA was obtained.

Preparation of Mn-BN and MnSA nanoparticle After fully trituration, Mn-BN flower/MnSA was collected and dispersed in anhydrous ethanol. The suspension was sonicated using a cell-disrupting probe sonicator (200 W, 20 kHz) to ensure complete dispersion. Following, DSPE-PEG$_{2000}$-NH$_2$ was fully mixture into the dispersion with Mn-BN/MnSA to achieve a homogeneous ethanol solution. And then, rotary evaporation was applied to remove the organic solvent. Finally, the final product was reconstituted in deionized water or saline to harvest the PEGylated Mn-BN /MnSA nanoparticle for further research.

Characterizations. X-ray powder diffraction (XRD) patterns were recorded on a Bruker D2 phaser with Cu Kα radiation at 30 kV. Fourier-transform infrared (FT-IR) spectra were measured with a Perkin Elmer Spectrum 100 system using a KBr disk method. Electron paramagnetic resonance (EPR) spectra were tested with a Bruker EMXplus-6/1 EPR spectrometer (center field: 3510.00 G; microwave frequency: 9.84 GHz; and microwave power: 6.325 mW) at room temperature. Scanning electron microscopy (SEM) was carried out with a QUATTRO S field-emission scanning electron microscope. Transmission electron microscopy (TEM) images were carried out with JEM-F200. The atomic-resolution high-angle annular dark-field scanning transmission electron microscopy (HAADF-STEM) image was obtained by a double aberration-corrected FEI Titan G2 60-300 S/TEM. X-ray photoelectron spectroscopy (XPS) measurements were performed on a Thermo Scientific ESCALAB Xi spectrometer with an Al Ka source, and the binding energies of all elements were calibrated through the C 1s peak (Binding Energy = 284.8 eV) as the standard. X-ray absorption spectroscopy (XAS) of Mn was performed at BL11B beamline at the Shanghai Synchrotron Radiation

Facility (SSRF). The data were collected from 6345 to 7323 eV and the energy was calibrated according to the absorption edge of a pure Mn foil. XANES and EXAFS data were processed using the Athena software package.

Cell lines. The murine 4T1 breast adenocarcinoma, Alpha Mouse Liver 12 (AML-12) and CT26`-Luc murine colorectal carcinoma cell lines were obtained from (Servicebio, China). 4T1, CT26 tumor cells was cultured in RPMI-1640 medium, and AML-12 cell was cultured in DMEM/F12 medium. Both of the culture media were supplemented with 10% fetal bovine serum (FBS; Gibco Life Technologies, California, USA) and 0.5% penicillin-streptomycin (Gibco Life Technologies, California, USA). Cells were maintained in a humidified incubator at 37°C under 5% CO_2, with media refreshed periodically to prevent confluency.

Cell viability. The cell viability was measured by a standard cell counting kit 8 (CCK-8) assay. Briefly, AML-12 cells were inoculated in 96-well plates at a density of 1×10^4 cells per well over night, and allowed to adhere overnight, and on the next day, the medium was replaced by the medium containing Mn-BN at different concentrations (0, 1.56, 3.16, 6.25, 12.5, 25, 50, 100, 200 μg/mL), and the incubation was continued for 24, 48 and 72 hours. Cell viability was subsequently studied by monitoring the absorbance of each well at 450 nm.

Cell uptake. Confocal microscopy analysis of Mn-BN cellular internalization in AML-12 and 4T1 cells. AML-12 and 4T1 cells were seeded at 5×10^4 cells/well in 35 mm glass-bottom dishes (NEST), respectively, and allowed to adhere for 24 hours. Prior to treatment, cells were serum-starved for 2 hours to minimize nonspecific endocytosis. The cells were incubated with Mn-BN PEGylated with DSPE-PEG_{2000}-FITC for different time periods (0.5, 1, 2, 4, 6 and 8 h). Subsequently, washed the

cells three times with PBS, before fixing with 4% paraformaldehyde for 20 minutes at room temperature, and followed by staining with DAPI for another 3 minutes. Following three more washes with PBS, the cells were analyzed using confocal laser scanning microscopy in conjunction with Zens (version 3.63) software and quantified the cell uptake by flow cytometry (CytExpert, Beckman Coulter).

Animal. Female BALB/c mice (6–8 weeks old, 16–20 g; Beijing Viton Lever, Beijing, China) were housed in individually ventilated cages (IVCs; 5 mice/cage) under controlled conditions: temperature (22 ± 2°C), humidity (50 ± 10%), and a 12-hour light/dark cycle. Food and water were provided ad libitum via automated systems. All experiments adhered to the National Institutes of Health Guide for the Care and Use of Laboratory Animals and the Chinese National Standard Laboratory Animal Welfare and Ethics (GB/T 35892-2018). All procedures adhered to relevant regulations and were reviewed and approved by the Peking University Cancer Hospital & Institute Animal Ethics Committee (Approval No. EAEC 2024-26).

Establishment of 4T1 cell subcutaneous tumor model in mice. Cells at 80–90% confluence were harvested using 0.25% trypsin-EDTA (Gibco Life Technologies, California, USA), washed twice with phosphate-buffered saline (PBS), and resuspended at a density of 1×10^6 cells/ 100 μL per mouse for further injection. 4T1 cells was subcutaneously injected into the right flank of six-week-old female BALB/c mice. Seven days later, when tumor volumes reached approximately 100 mm^3, the mice were randomly divided into $MnCl_2$ and Mn-BN groups. After obtained the baseline images before injected contrast, the post-injected MRI enhancement images were obtained from different time points with different concentration of manganese-based contrast.

Establishment of CT26-Luc cell-derived liver metastasis model in mice. Cells at 80-90% confluence were harvested using 0.25% trypsin-EDTA (Gibco Life Technologies, California, USA), washed twice with phosphate-buffered saline (PBS), and resuspended at a density of 5×10^6 cells/ 100 μL per mouse for injection. Six-week-old female BALB/c mice were anesthetized via intraperitoneal injection of ketamine/xylazine (100/10 mg/kg). A 30-gauge needle was inserted into the splenic parenchyma at a shallow angle, and prepared CT26-Luc cells suspension were slowly injected to minimize intraperitoneal leakage. The establishment of liver metastasis was tracked using an in vivo imager (IVIS Spectrum System, PerkinElmer Ltd.).

In vitro MRI. The phantom MRI were performed on a 3T clinical MRI system (United Imaging, UMR790) with mouse coil for the Mn-BN, Mn-DPDP, $MnCl_2$ in aqueous solution with different concentration to acquire the T1 and T2 weighted imaging and the relaxivity with T1 mapping and T2 mapping. The parameters for T1WI at 3T were repetition time (TR) of 500 ms, echo time (TE) of 13.28 ms, the field of view (FOV) of 70 mm × 30 mm, slice thickness of 5 mm, and Matrix of 336 × 100. The parameters for T2WI at 3T were TR of 3000 ms, TE of 106.24 ms, the FOV of 70 mm × 30 mm, slice thickness of 5 mm, and Matrix of 336 × 100. The T1 mapping parameters were TR of 10000 ms, TE of 39.04 ms, the FOV of 70 mm × 30 mm, slice thickness of 5 mm, and Matrix of 160 × 100 and inversion time (TI) = 100 + 150n ms, where n = 0, 1, 2, ..., 19. The T2 mapping parameters were TR of 10000 ms, the FOV of 70 mm × 30 mm, slice thickness of 5 mm, and Matrix of 176 × 100 and TE = 20 + 20n ms, where n = 0, 1, 2, ..., 32. The relaxivity and MRI phantom images of MN-BN were also recorded at 7 T animal MRI system (Bruker, Biospin 7T PharmaScan7016) . The parameters for T1WI at 7T were TR of 200 ms, TE of 8 ms, FOV of 30 mm × 30 mm, slice thickness of 0.8 mm, and Matrix of 256 × 256. The parameters for T2WI at 7T were TR of 2200 ms, TE of 8

ms, the FOV of 30 mm × 30 mm, slice thickness of 0.8 mm, and Matrix of 192 × 192. The T1 mapping parameters were TE of 8.5 ms, the FOV of 30 mm × 30 mm, slice thickness of 0.8 mm, and Matrix of 192 × 256 and TR = 400, 800, 1500, 3000, 6000, 12000 ms. The T2 mapping parameters were TR of 2200 ms, the FOV of 30 mm × 30 mm, slice thickness of 0.8 mm, and Matrix of 192 × 192 and TE = 8n ms, where n = 1, 2, 3, ..., 12. The relaxivity was analyzed with uWS-MR Vision R005.4.0.

In vivo MRI. All in vivo MRI were performed on a 3T clinical MRI system (United Imaging, UMR790) with mouse coil. For T1 weighed imaging, axial and coronal images were obtained before and different periods post intravenous injection of Mn-BN and $MnCl_2$ for tumor - bearing mouse. For coronal T1WI, the scanning sequence parameters were TR of 543 ms, echo time TE of 12.54 ms, the field of view FOV of 80 mm × 40 mm, slice thickness of 1.5 mm, and Matrix of 352 × 100. The parameters of axial T1WI sequence in subcutaneous tumor scanning were TR of 508 ms, TE of 12.62 ms, FOV of 72 mm × 40 mm, slice thickness of 1.5 mm, and Matrix of 320 × 100. The parameters of axial T1WI sequence in liver metastases tumor scanning were TR of 400 ms, TE of 12.92 ms, FOV of 72 mm × 40 mm, slice thickness of 1.1 mm, and Matrix of 320 × 100.

Hemolysis test. Freshly collected mouse blood in anticoagulated tubes was subsequently centrifuged to obtain erythrocyte precipitates. The collected blood cells were resuspended in saline and adjusted to the 4% concentrated solution. Triton solution and saline were used as positive and negative controls, respectively, and Mn-BN nano contrast agents with a concentration gradient were set up for co-culture with the hemocyte solution. After incubation, all blood samples were centrifuged, and the supernatant was collected and the absorbance at 540 nm was measured. The percentage of hemolysis was calculated according to the following formula:

$$\text{Hemolysis (\%)}=\left[\frac{Ab_{sample}-Ab_{negativecontrol}}{Ab_{positvivecontrol}-Ab_{negativecontrol}}\right]\times 100\%$$

In vivo biosafety evaluation of Mn-BN. Healthy female mice were intravenously administered Mn-BN or MnCl2 (Mn 360 μg/kg, triple the standard dose), designated as experimental and control cohorts (n = 4 per group). On day 7 post-administration, major organs (brain, heart, liver, spleen, lungs, and kidneys) were harvested, immersed in 4% buffered paraformaldehyde solution, and processed for hematoxylin and eosin (H&E) staining. Histological examination of stained tissue sections was conducted using a light microscope (Nikon Eclipse E600, Nikon Inc.).

Blood samples were collected for serum biochemical profiling. Harvested serum was subjected to centrifugation at 15,000 rpm for 10 minutes, and the resultant supernatant was retained for analysis. Aminotransferase (ALT), albumin (ALB), creatinine (CREA), blood urea nitrogen (BUN) alkaline phosphatase (ALP), creatine kinase (CK), and aspartate aminotransferase (AST) were quantified in the serum employing an automated biochemical analyzer (Hitach, 7180). Concurrently, hematology analysis including white blood cells (WBCs), red blood cells (RBCs), hemoglobin (HGB) and platelets (PLT) in blood were assessed utilizing a fully automated hematology analyzer (Sysmex, XN-1000V).

Statistical analysis. For statistical analyses, all in vitro and in vivo experiments were conducted in triplicate, with results expressed as the mean ± SD. Data analysis and visualization were performed using GraphPad Prism software (version 9.5.0). Comparisons between two groups were made using Student's t-test, while differences among three or more groups were assessed using one-way or two-way analysis of variance (ANOVA). Error bars represent the standard deviation of three, four, or five independent measurements. Imaging experiments were repeated three times with consistent results.

Statistically significant P-values are indicated in the figure legends. *$p < 0.05$, **$p < 0.01$, ***$p < 0.001$, and ****$p < 0.0001$ were considered statistically significant.

Calculations methods. Molecular geometry optimization calculations were carried out using the CP2K 2024.1 program[1], which employs a Gaussian/plane wave hybrid basis set (GPW). The Perdew-Burke-Ernzerhof (PBE) functional[2] was adopted together with Grimme's D3(BJ) dispersion correction parameters[3, 4] to improve calculation accuracy. The DZVP-MOLOPT basis set and Goedecker-Teter-Hutter (GTH) pseudopotentials[5, 6] were utilized in the computations. The plane wave function cutoff energy and reference cutoff energy were set to 400 Ry and 55 Ry, respectively, to ensure energy convergence. The Broyden-Fletcher-Goldfarb-Shanno (BFGS) algorithm was applied for structural optimization, with the termination criterion being that the force on each atom is less than 0.00045 Hartree/Bohr. For self-consistent total energy calculations, the convergence criterion was set to 1×10^{-5} Hartree. In the case of the $Mn_2O_3N_3$ system, the number of k-points in the three directions of the MONKHORST-PACK grid was $3 \times 3 \times 1$. During single-point energy calculations, the plane wave function cutoff and reference cutoff energies were adjusted to 600 Ry and 55 Ry, respectively. The TZVP-GTH basis set and GTH pseudopotentials[5, 6] were used for C, H, O, N, B, and Cl atoms, while the TZVP-MOLOPT-PBE-GTH basis set and GTH pseudopotentials[5, 6] were employed for Mn atoms. The adsorption energy (ΔE) was defined as the difference between the energy of the complex and the sum of the energies of the monomers, described by the formula $\Delta E = E(A+B) - E(A) - E(B)$. All wave function analyses were completed using the Multiwfn 3.8 (dev) code[7, 8].

Supporting Figures

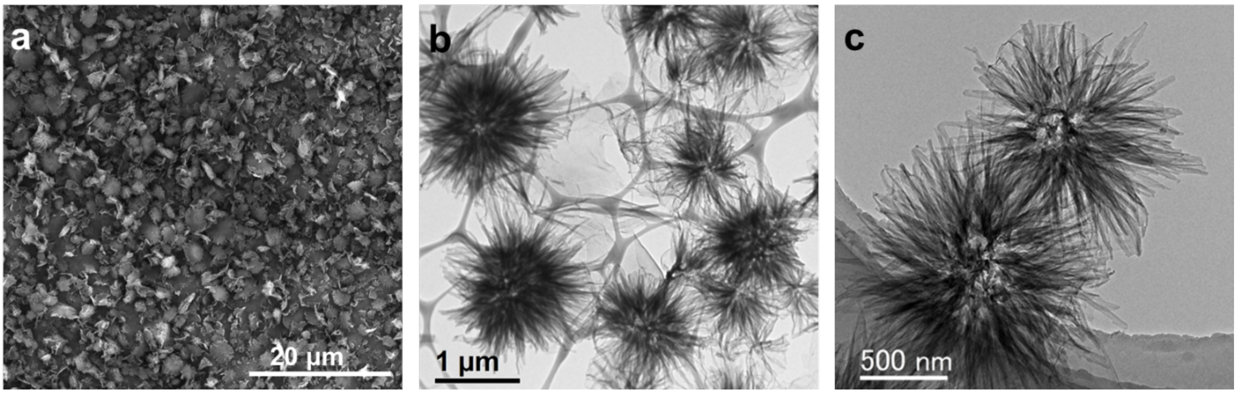

Figure S1. (a) SEM image and (b-c) TEM images of Mn-BN.

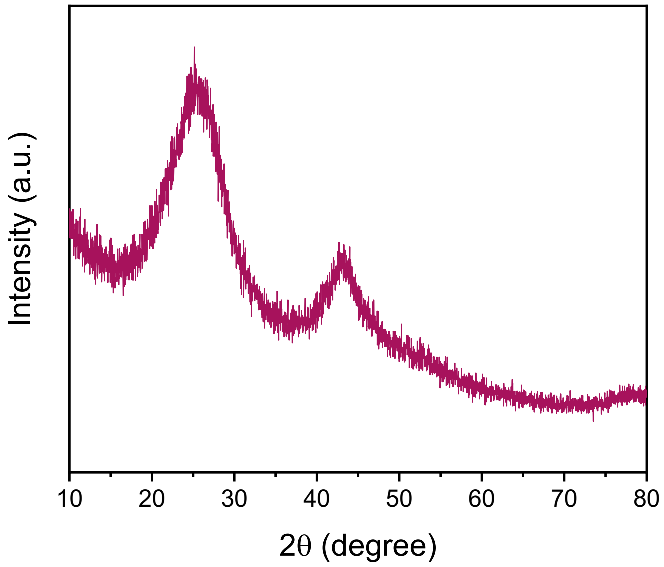

Figure S2. XRD pattern of Mn-BN.

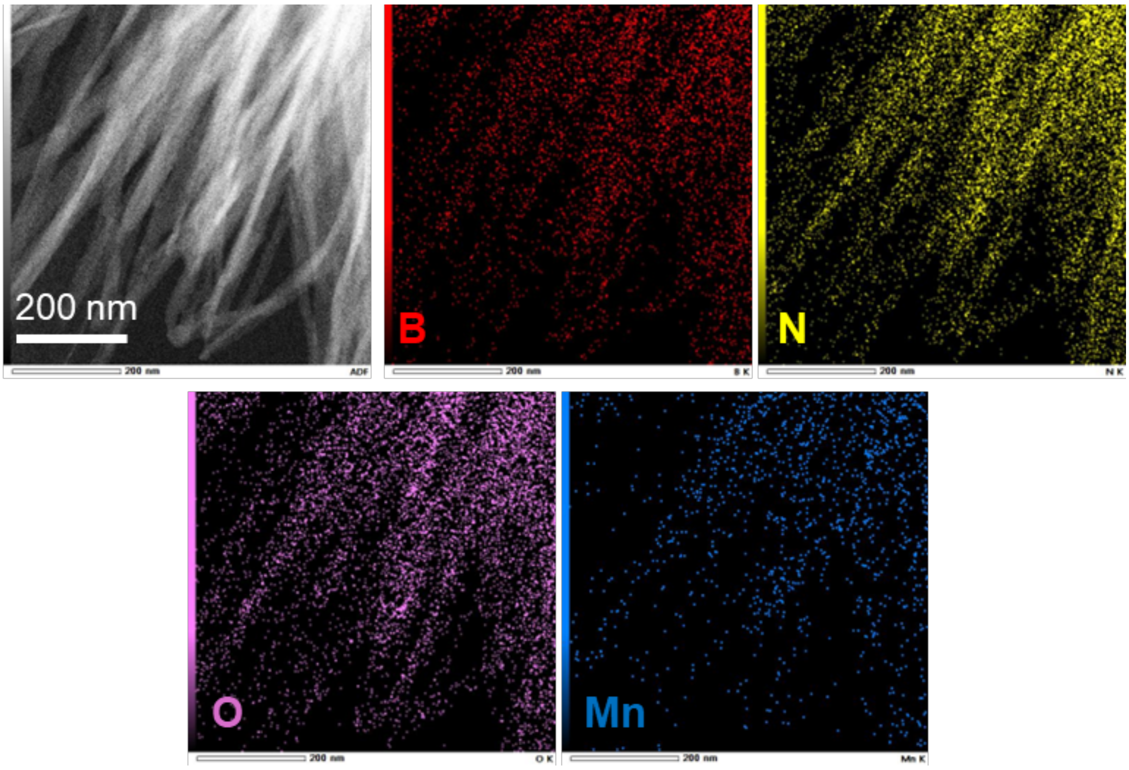

Figure S3. TEM elemental mapping images of Mn-BN flower.

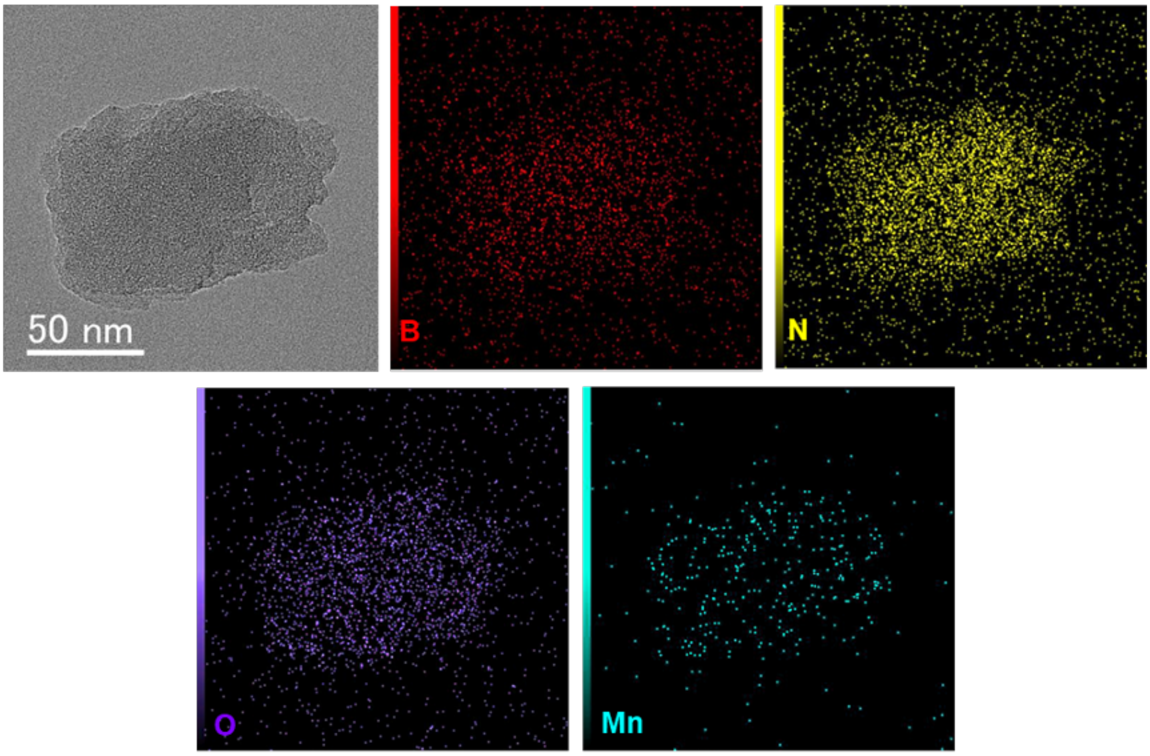

Figure S4. TEM elemental mapping images of Mn-BN nanoflakes.

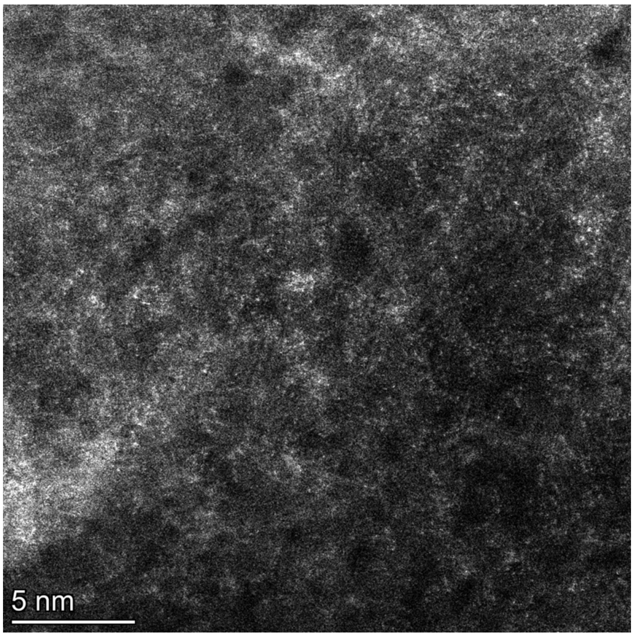

Figure S5. Aberration-corrected HAADF-STEM image of Mn-BN nanoflakes.

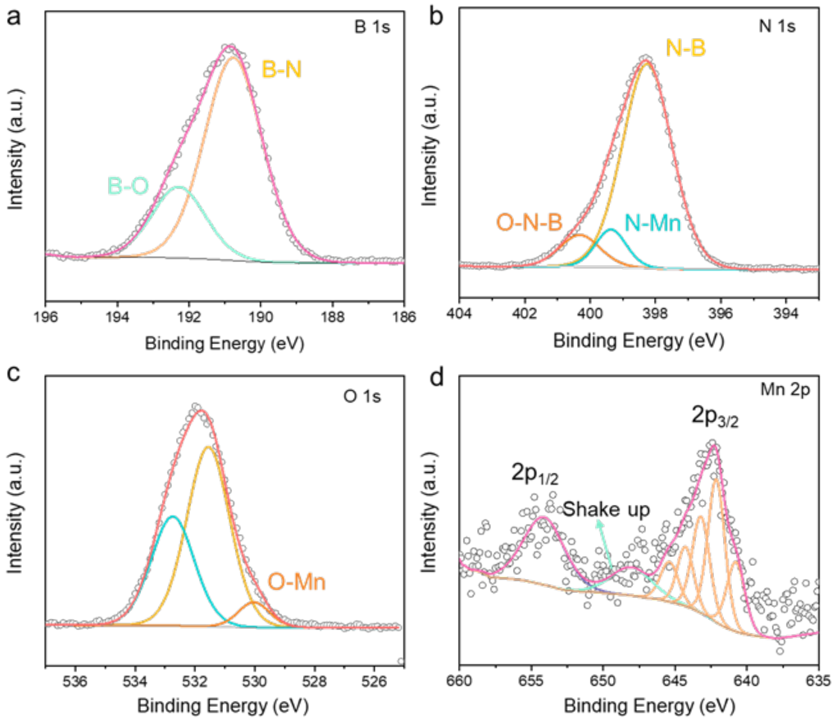

Figure S6. High resolution XPS spectra of B 1s, N 1s, O 1s, and Mn 2p for Mn-BN flower.

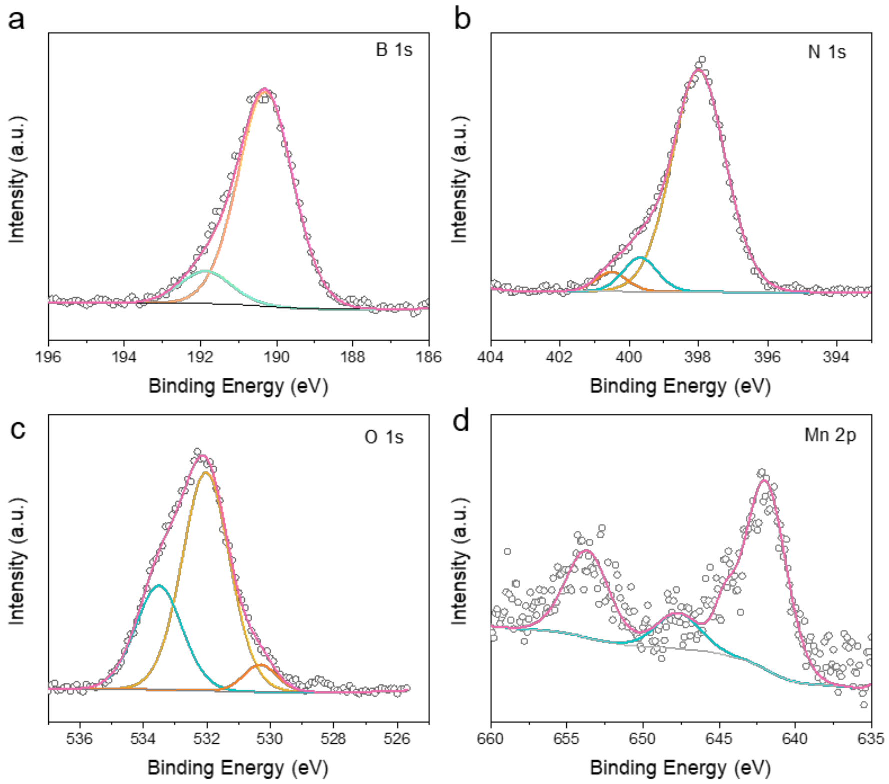

Figure S7. High resolution XPS spectra of B 1s, N 1s, O 1s, and Mn 2p for Mn-BN nanoflakes.

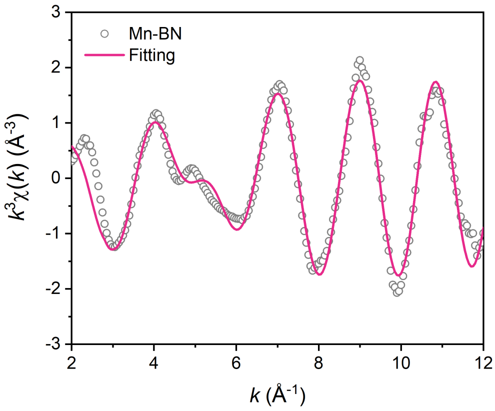

Figure S8. EXAFS spectra fitting in K space of Mn-BN flower.

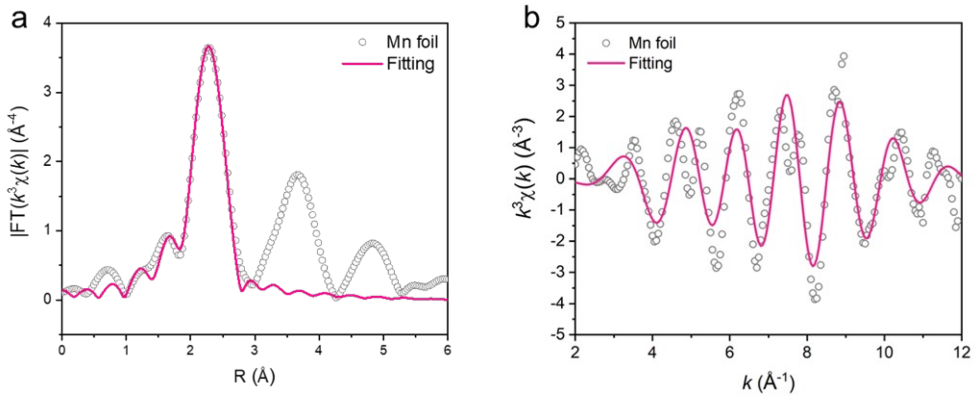

Figure S9. EXAFS fitting curve of Mn foil in *R* and *K* space.

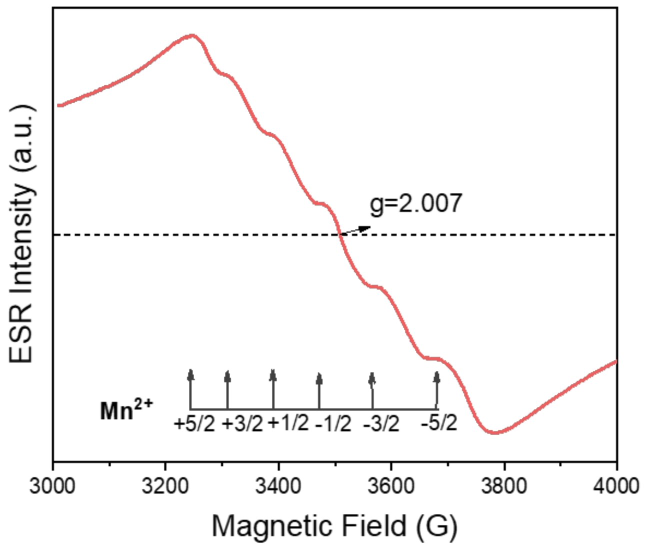

Figure S10. ESR spectrum of Mn-BN flower, g value is 2.007.

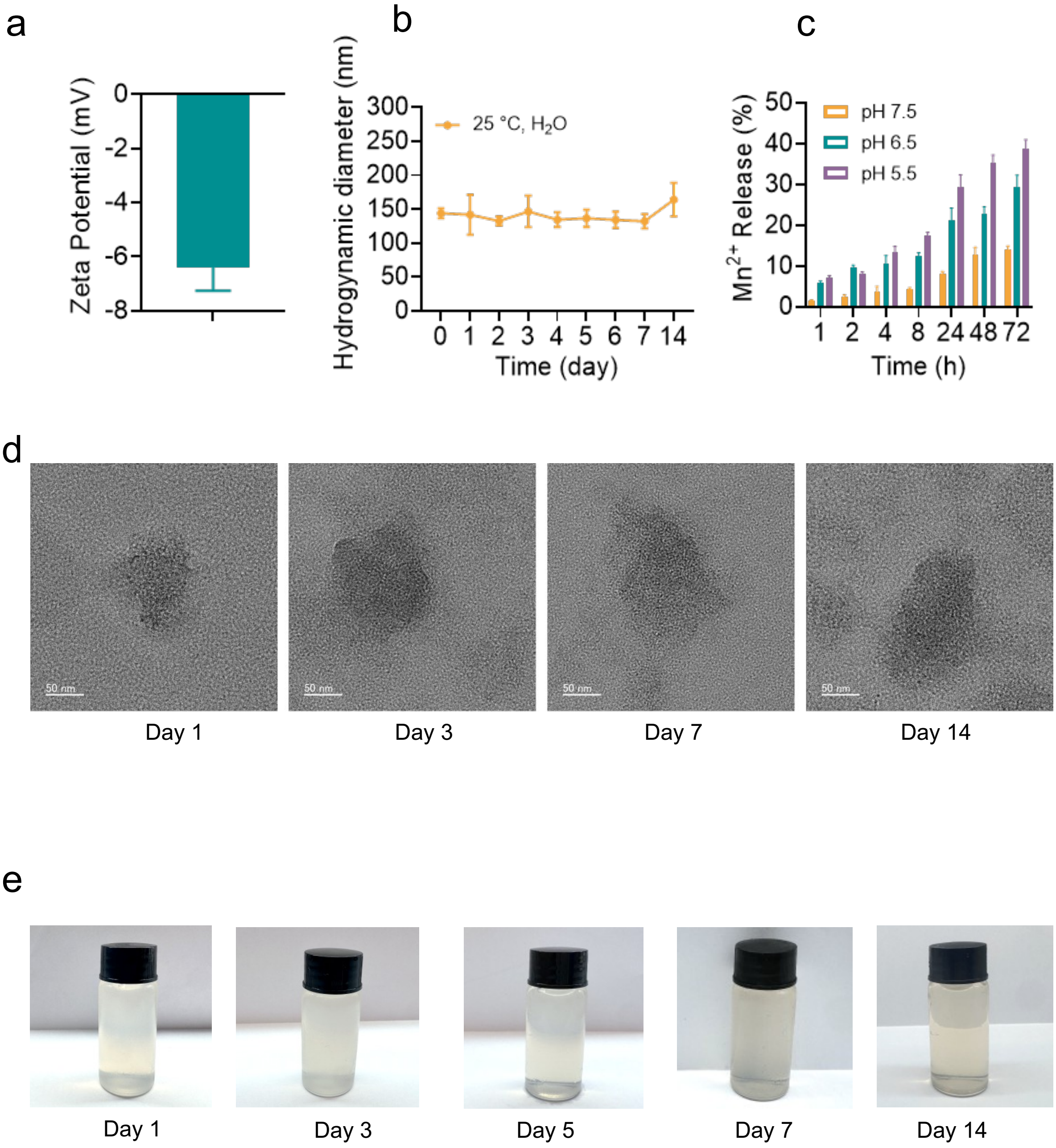

Figure S11. The character and stability of PEGylated Mn-BN nanoparticles with all statistical data as mean values ± SD (a)Zeta potential; (b) the DLS; (c) Quantitative ICP-MS analysis for free of Mn^{2+} release at pH 5.5, 6.5, and 7.5. (d) TEM images (e) photos of aqueous solution.

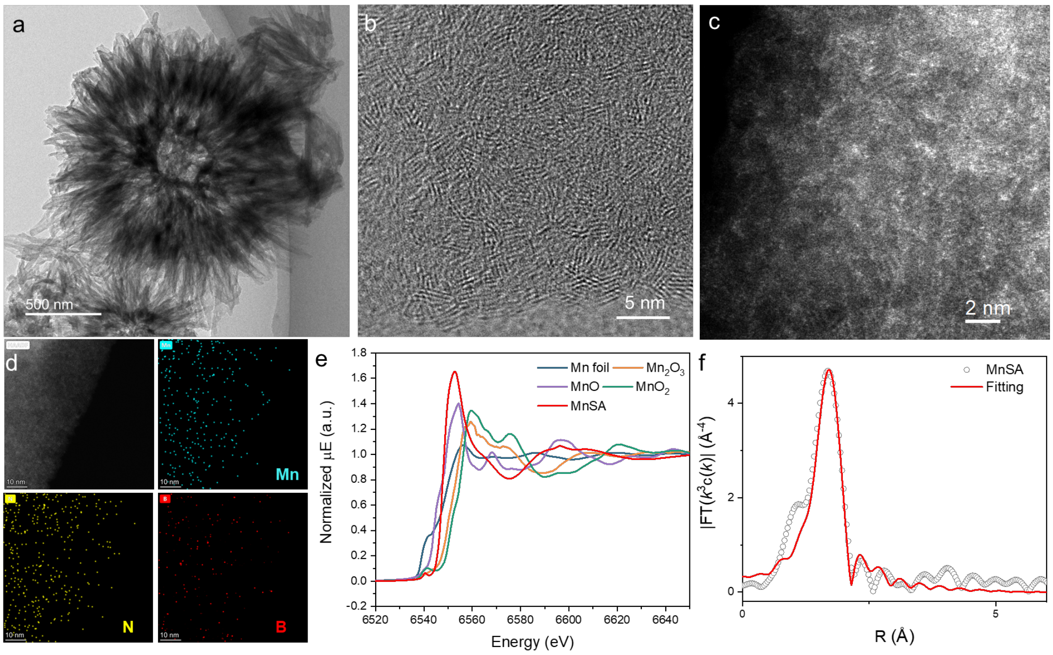

Figure S12. (a) TEM image, (b) HRTEM image, (c) AC-HAADF-STEM image, (d) corresponding high-resolution elemental mapping images, (e) Mn K-edge XANES of MnSA, and (f) EXAFS fitting curve of MnSA in R space.

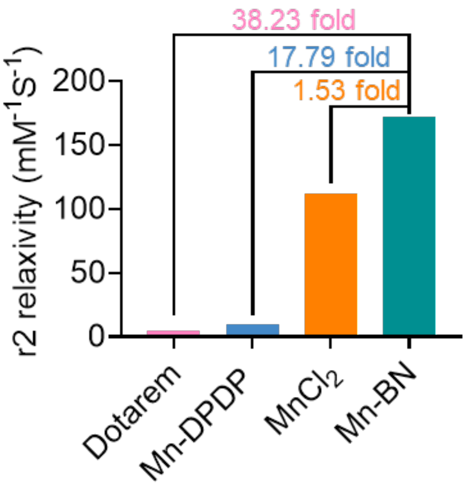

Figure S13. Comparison of r2 relaxivities of Dotarem, Mn-DPDP, $MnCl_2$ and Mn-BN at 3T.

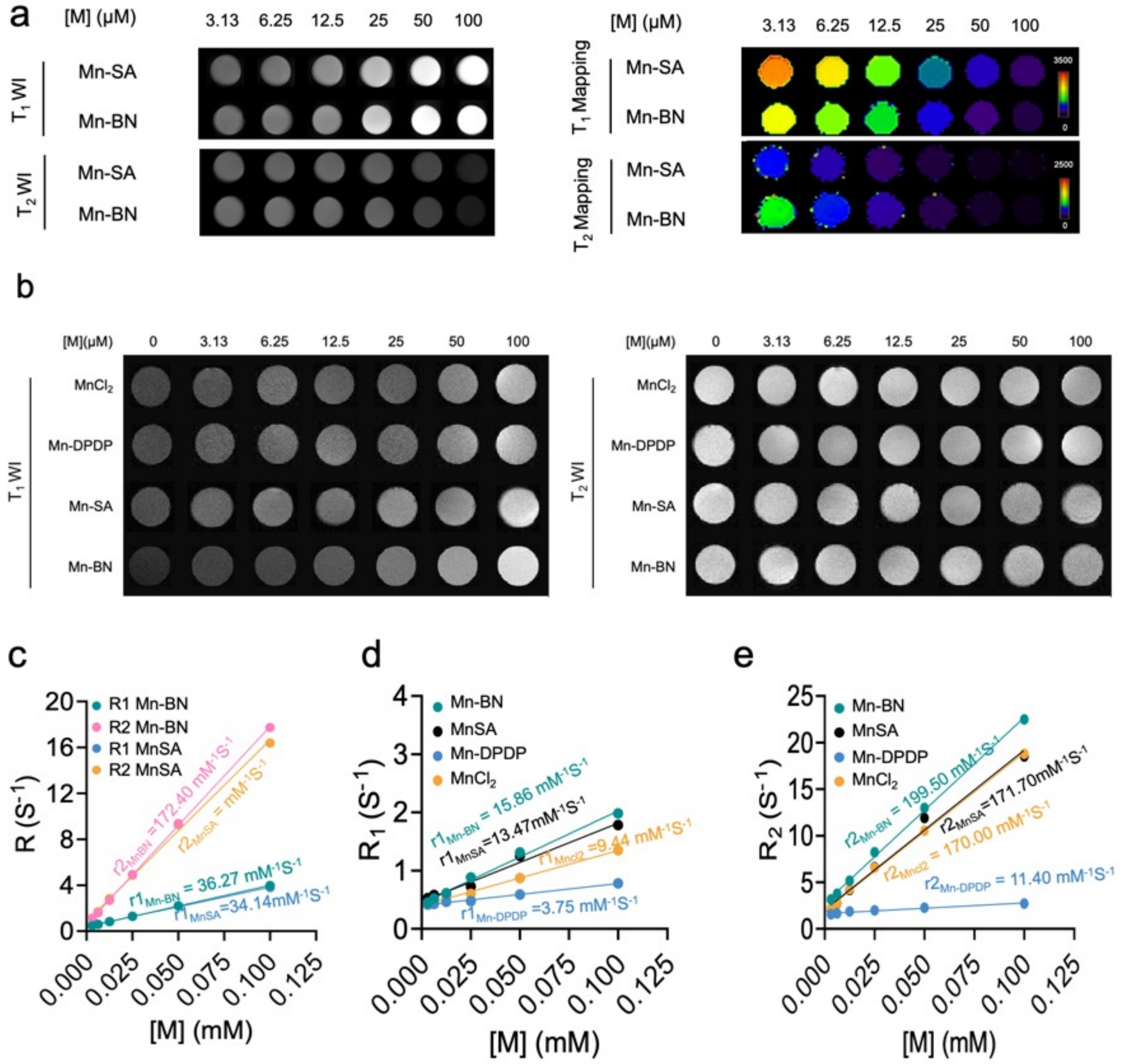

Figure S14. (a) Phantom MRI of MnSA and Mn-BN in aqueous solution with different metal concentrations at 3T (b) T1WI and T2WI phantoms of Mn-BN, Mn-DPDP, $MnCl_2$, and MnSA in aqueous solution with different metal concentrations at 7T; Metal concentration-dependent corresponding relaxation time R1 and R2 of MnSA and Mn-BN at 3T; (c)Metal concentration-dependent corresponding relaxation time R1(d) and R2(e) at 7T.

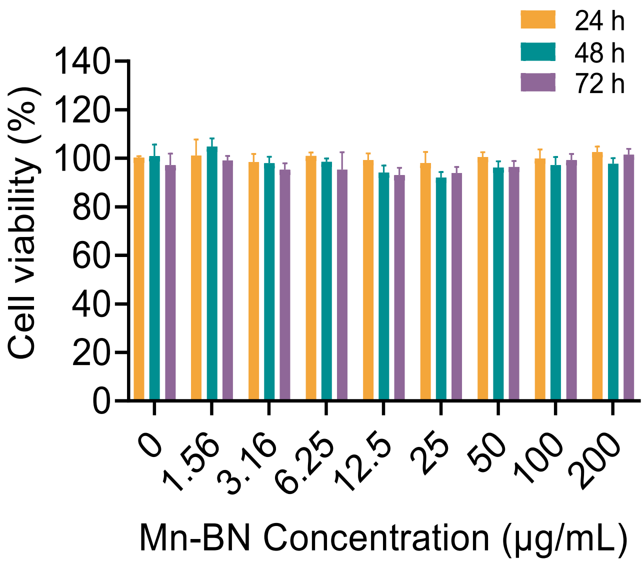

Figure S15. Cell viability of AML-12 cells with incubation of different concentration of Mn-BN for 24, 48 and 72 hours. No observable cytotoxicity in AML-12 cell. All statistical data as mean values ± SD (n=4).

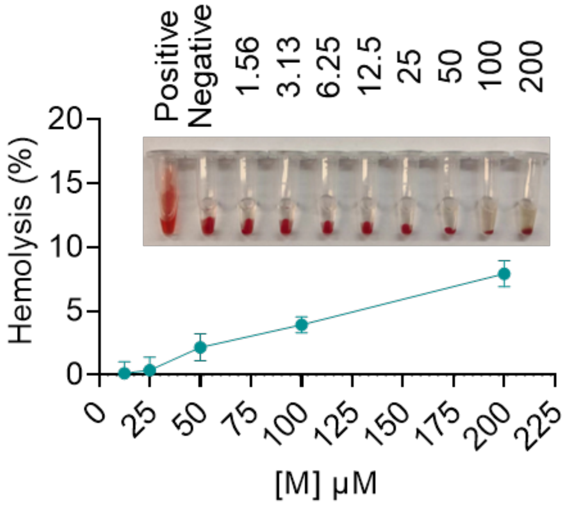

Figure S16. Hemolysis of different concentrations of Mn-BN-treated, with negligible hemolytic activity below 100 μM Mn concentration.

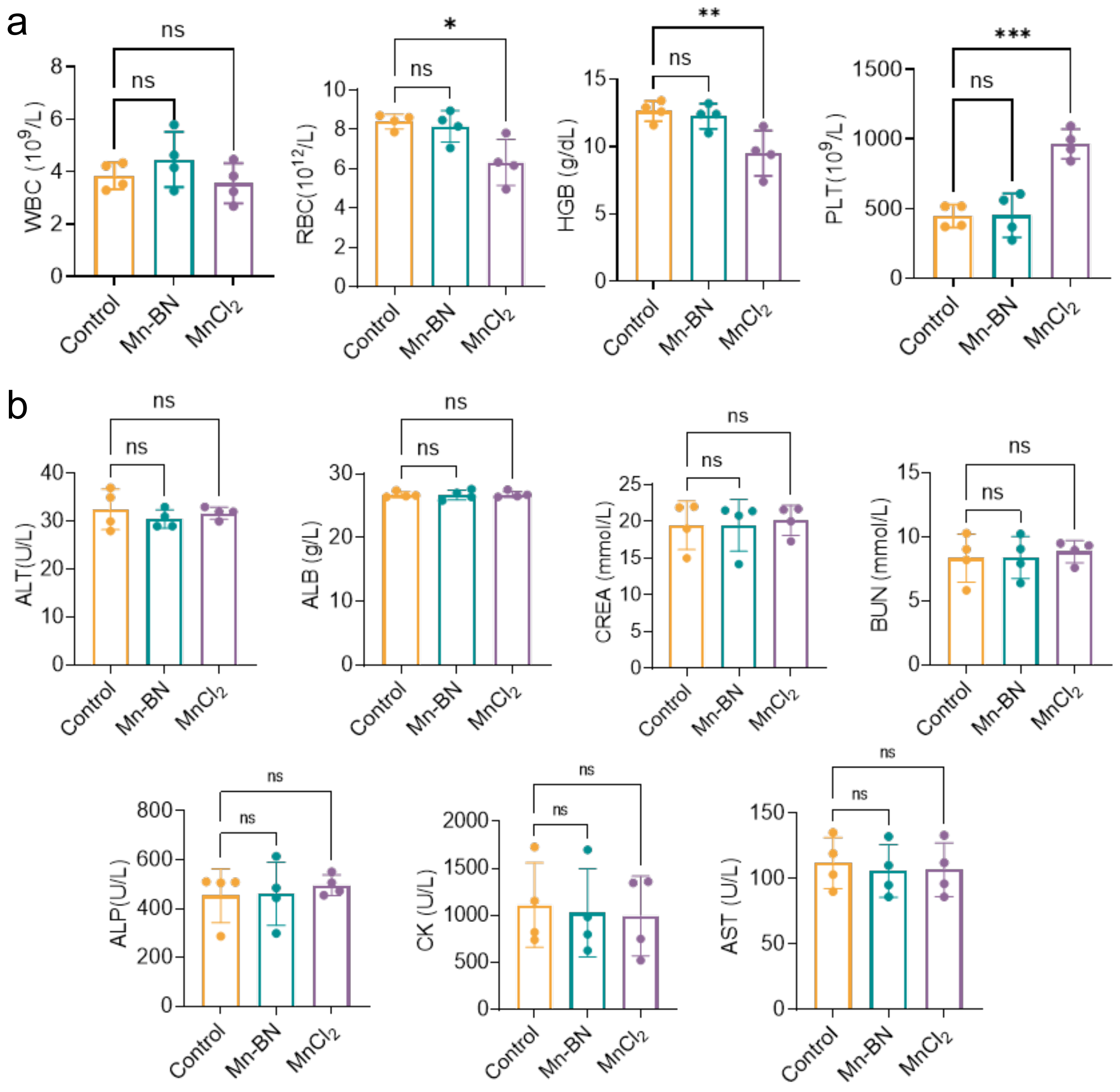

Figure S17. In vivo biosafety assessments of Mn-BN or $MnCl_2$ in healthy mice (n = 4) with all statistical data as mean values ± SD. (a) Hematology analysis of counts of white blood cells (WBCs), red blood cells (RBCs), hemoglobin (HGB) and platelets (PLT) in blood; the mice in the $MnCl_2$ group shows significant abnormality of blood indexes among RBC, HGB, PLT at the same dose induced. (b)Blood biochemistry analysis of concentration of aminotransferase (ALT), albumin (ALB), creatinine (CREA), blood urea nitrogen (BUN), alkaline phosphatase (ALP), creatine kinase (CK) and aspartate aminotransferase (AST).

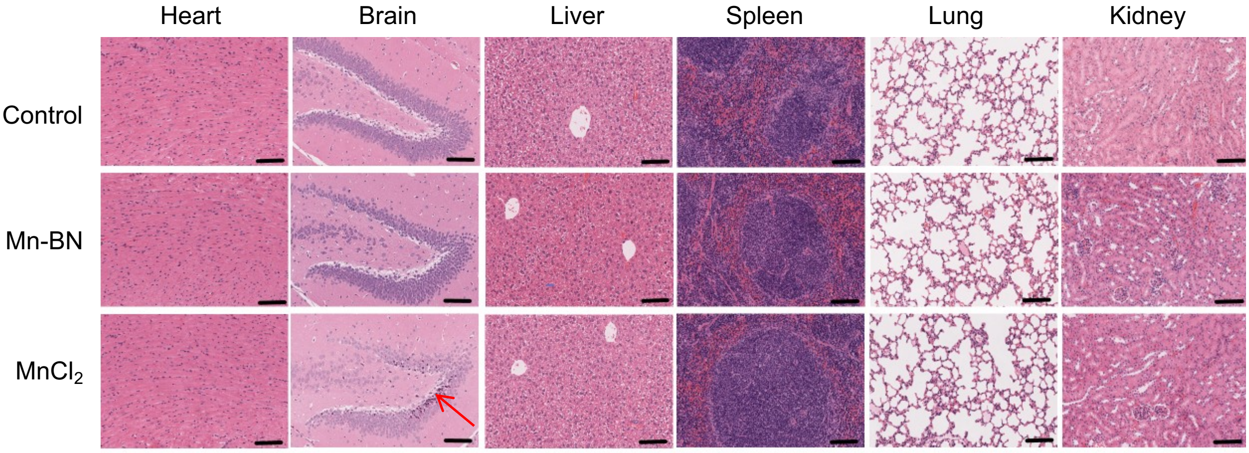

Figure S18. In vivo biosafety assessments in healthy mice of Mn-BN or $MnCl_2$ group H&E staining of major organs, including heart, brain, liver, spleen, lung, and kidney. In the $MnCl_2$ group, the presence of red neurons, which shows pyknosis (nuclear condensation) and eosinophilic (red-stained) cytoplasm marked with red arrow. Scale bar: 100 µm.

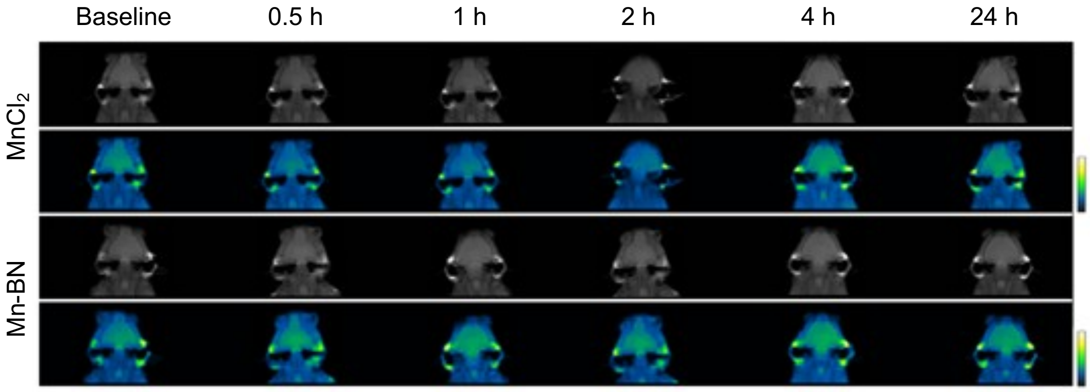

Figure S19. Contrast enhanced T1 weighted images of head of BALB/c mice after intravenous injection of 120 µg/kg Mn per mouse of Mn-BN and $MnCl_2$ for 24 hours.

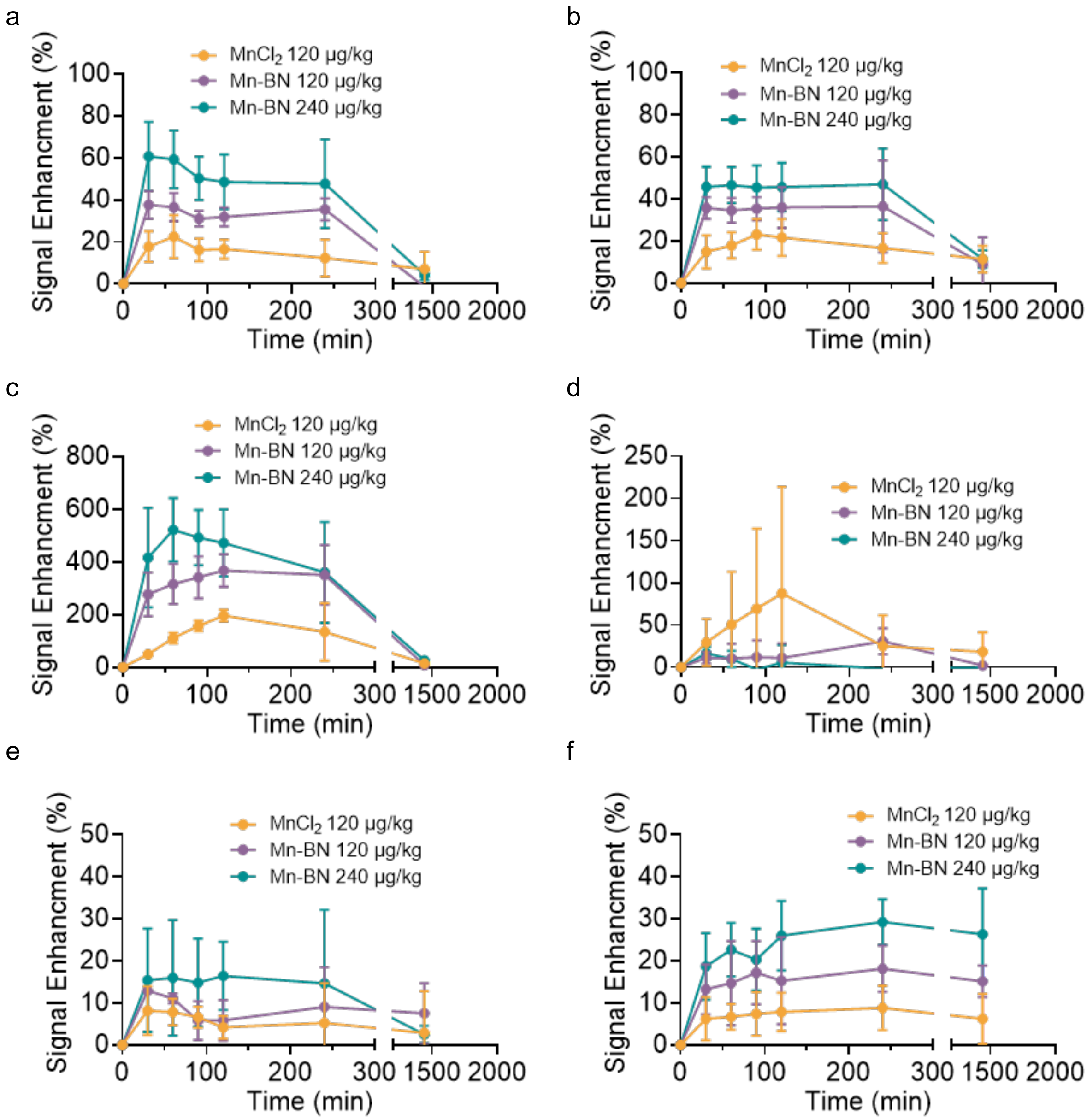

Figure S20. Contrast enhanced MRI signal in different organs of BALB/c mice bearing subcutaneous tumor (4T1 cells) in 24 hours with Mn-BN and $MnCl_2$ intravenous injection at 3T (n = 3 for each group) with all statistical data as mean values ± SD, a) liver; b) kidney; c) gall bladder; d) bladder; e) spleen; and f) tumor.

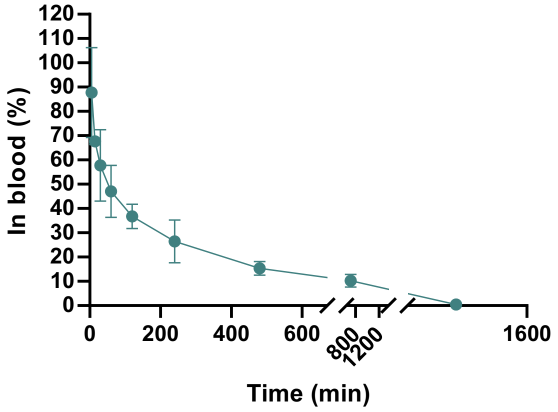

Figure S21. The Mn-BN-Cy5 (120 μg/kg Mn) remaining in the blood at different time points post-intravenous injection (n = 3).

a

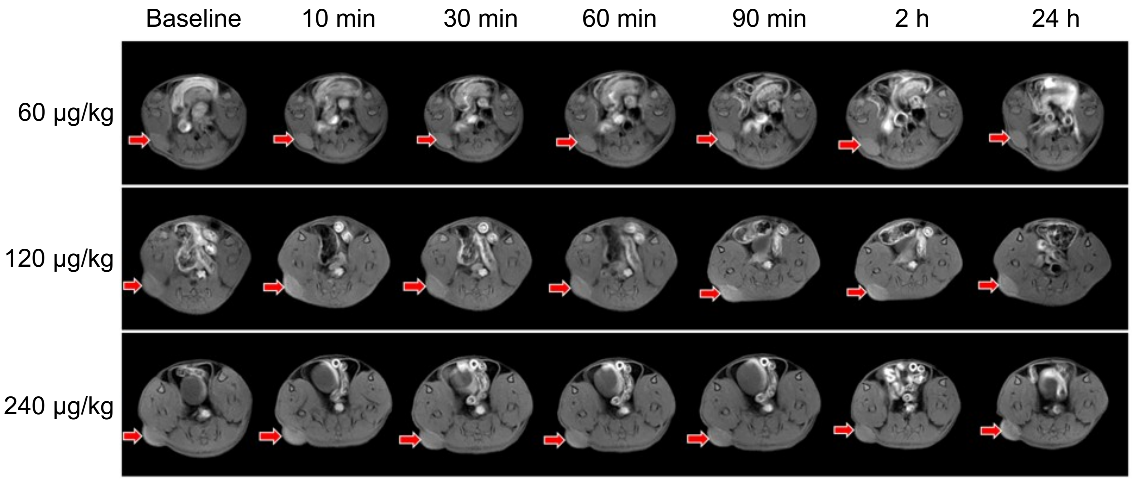

b

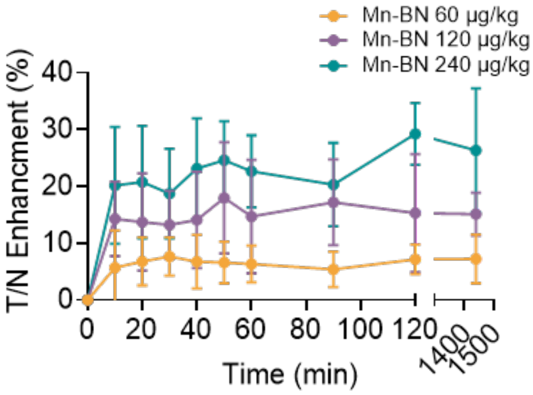

Figure S22. Contrast enhanced MRI of BALB/c mice bearing subcutaneous tumor (4T1 cells) with different concentration of Mn-BN (60, 120, 240 μg/kg Mn per mouse) at 3T. a) T1-weighted MR images acquired at different time points after intravenous injection of Mn-BN, b) Corresponding T1 signal enhancement of tumor in dose different groups (n = 3 for each group).

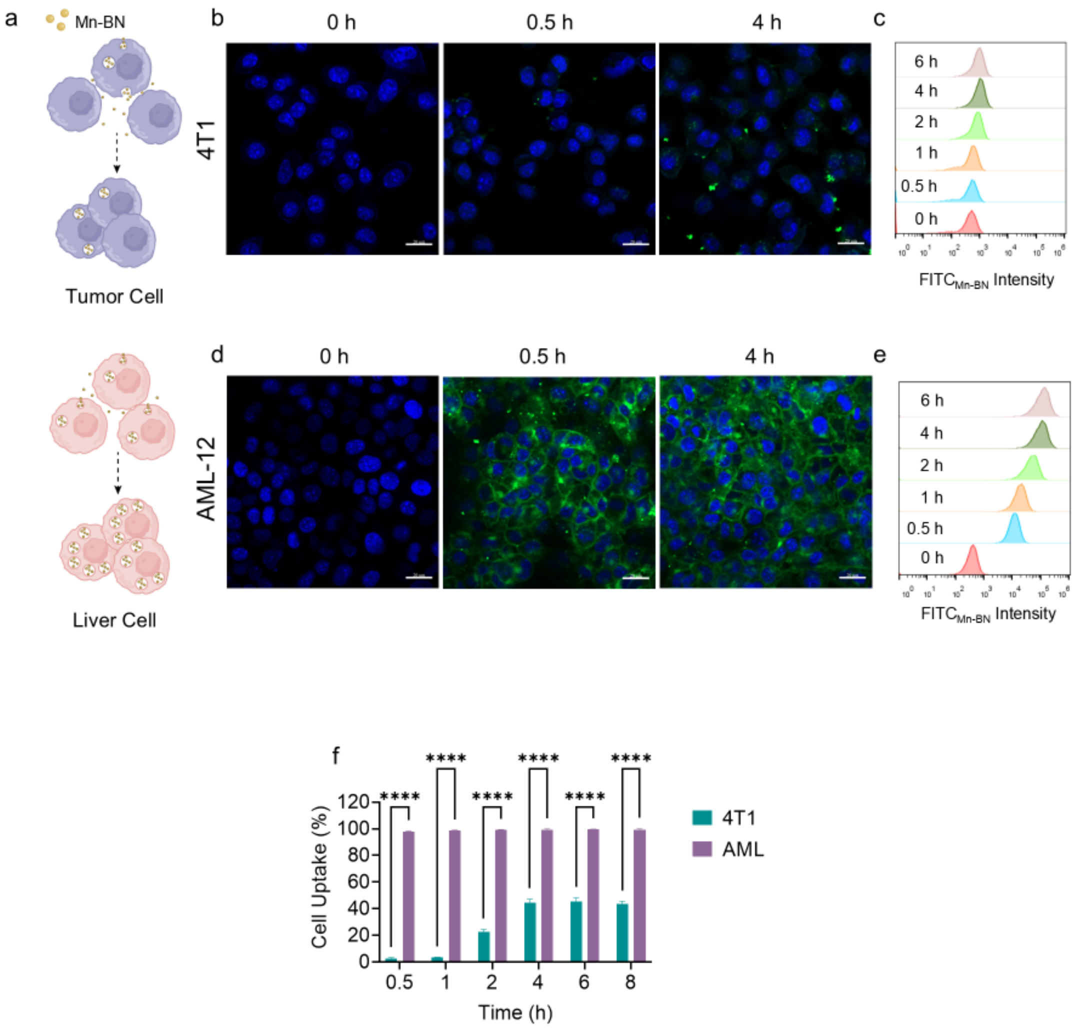

Figure S23. (a) Schematic illustration of the cell uptake of Mn-BN in tumor cells and liver cells; (b-f) Confocal microscopy images and flow cytometry analysis of intracellular uptake after incubation with Mn-BN PEGylated with DSPE-PEG$_{2000}$-FITC for different time points (0, 0.5, 1, 2 and 4 h) in vitro, b,c) 4T1 cells and d,e) AML-12 cells. Scale bar = 20 μm, n = 3; f) flow cytometry analysis at the different time points (0, 0.5, 1, 2, 4, 6 and 8 h).

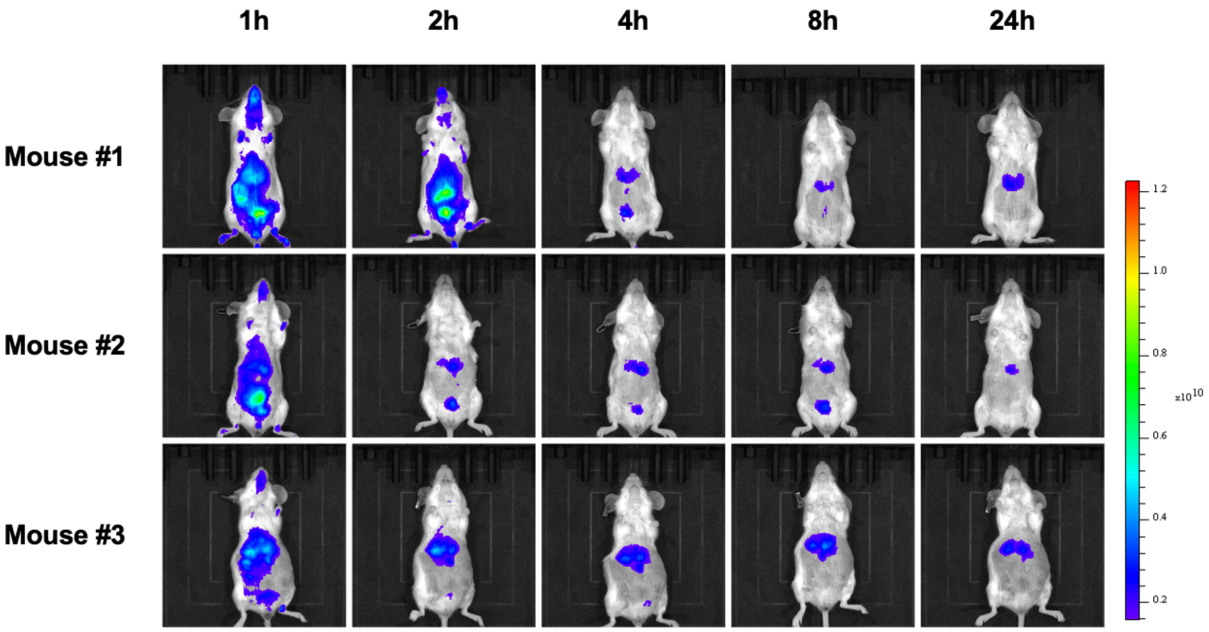

Figure S24. In vivo fluorescence imaging of BALB/c mice bearing liver metastases (CT26-luc cells) with Mn-BN-Cy5 (120 μg/kg Mn) at different time points (n = 3).

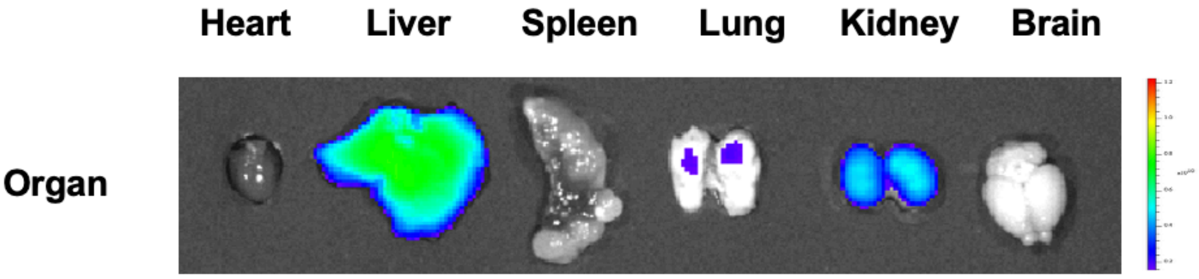

Figure S25. Ex vivo fluorescent imaging of isolated organs BALB/c mice bearing liver metastases (CT26-luc cells) with Mn-BN-Cy5 (120 μg/kg Mn) at 24 hours after treatment.

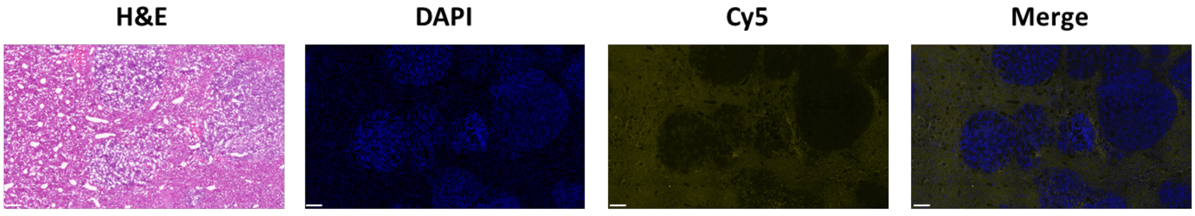

Figure S26. In situ assessment of Mn-BN nanoparticles, histological and fluorescence imaging of liver metastases tissues after 8 hours injection of Mn-BN-Cy5. Scale bar 200 µm.

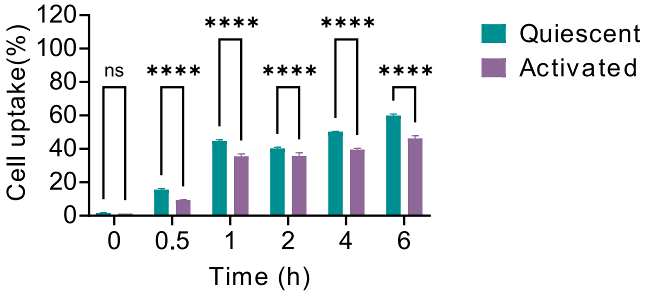

Figure S27. Flow cytometry analysis of intracellular uptake after incubation LX-2 cells with Mn-BN PEGylated with DSPE-PEG$_{2000}$-FITC for different time points at different time points (0, 0.5, 1, 2, 4, and 6h).

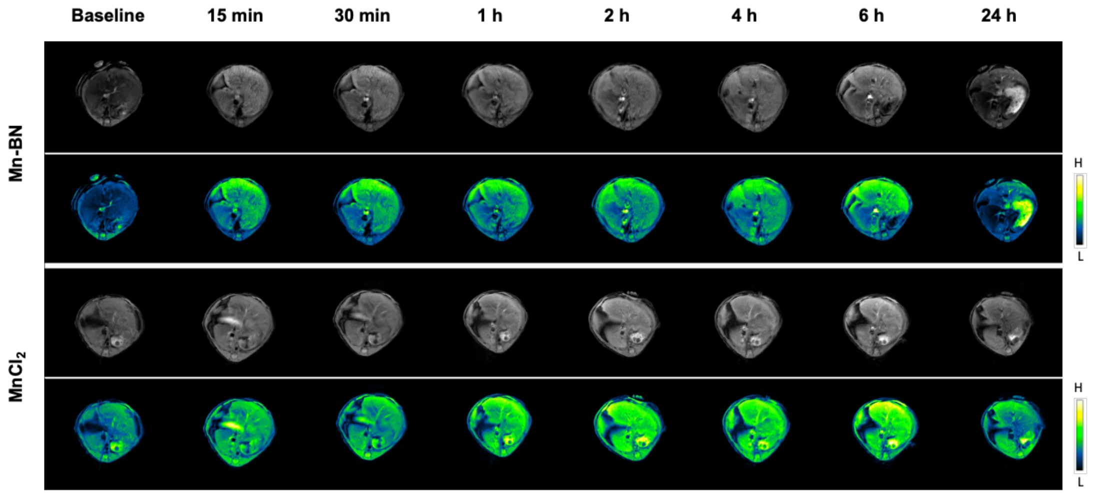

Figure S28. Contrast-enhanced MRI of mice bearing liver fibrosis with Mn-BN and $MnCl_2$ at 3T. Axial T1-weighted MR images acquired at different time points after intravenous injection of Mn-BN or $MnCl_2$ (120 µg/kg Mn per mouse), T1-weighted axial upper abdominal images were acquired using a clinical 3T MRI scanner (United Imaging, UMR790).

Table S1. EXAFS fitting results of Mn-BN, Mn foil and MnO_2.

	Path	R(Å)	C.N.	ΔE0 (eV)	σ^2 (Å^2)	R-factor
Mn foil	Mn-Mn	2.33	3		0.04	
	Mn-Mn	2.58	3	-11.332	0.05	0.025
	Mn-Mn	2.95	2		0.08	
Mn-BN	Mn-N	1.92	2.1		0.003	
	Mn-O	2.16	1.8	-2.81	0.005	0.007
	Mn-Mn	2.39	0.9		0.032	
Mn-SA	Mn-N	2.18	4	2.25	0.008	0.029

Note: S_0^2 = 0.80, R is the bond length, CN is the coordination number around the Mn absorber; σ^2 is the Debye-Waller factor (a measure of thermal and static disorder in absorber-scatterer distances); ΔE0 is edge-energy shift (the difference between the zero kinetic energy value of the sample and that of the theoretical model); R-factor reflects the quality of the fitting. Error bounds that characterize the structural parameters obtained by EXAFS spectroscopy were estimated as CN ±20%; R ± 2%; σ2 ± 20%; ΔE0 ± 20%.

Table S2. Relaxivity of manganese-based contrast agents.

Agent	r1 ($mM^{-1}s^{-1}$)		r2 ($mM^{-1}s^{-1}$)	
	3T	7T	3T	7T
**Mn-BN**	**36.27**	**15.86**	**172.40**	**199.50**
Mn-Cl_2	7.32	9.44	112.40	170.00
Mn-DPDP	2.86	3.75	9.69	11.40
MnSA	34.14	13.47	158.20	171.70

Table S3. Comparison of relaxivity of Mn-BN with commercial contrast agents and conventional Mn-based contrast agents.

Cat.	No.	Material	Relaxivity (mM^{-1} s^{-1})	Field	Refs
Single atom agents	1	**Mn-BN**	**r1 = 36.27**<br>**r2 = 172.40**	**3.0 T**	**This work**
			**r1 = 15.86**<br>**r2 = 199.50**	**7.0 T**	
	2	feGd-NxC	r1 = 65.5<br>r2 = 74.2	0.5 T	9
			r1 = 34.2<br>r2 = 80.10	3.0 T	
			r1 = 12.0<br>r2 = 113.50	7.0 T	
	3	Fe-SANBs	r1 = 16.80	0.5 T	10
			r1 = 11.48	3.0 T	
			r1 = 4.73	7.0 T	
	4	Gd-SA	r1 = 11.05<br>r2 = 102.80	7.0 T	11
	5	R-Mn-CDs	r1 = 5.82	/	12
Commercial contrast agents	6	Mn-DPDP	r1 = 2.8<br>r2 = 3.7	1.5 T	13
	7	LumenHance ($MnCl_2$)	r1 = 8.96<br>r2 = 93.83	1.5 T	14
			r1 = 6.97<br>r2 = 116.5	3.0 T	
	8	Gd-BOPTA	r1 = 8.1<br>r2 = 18.7	1.5 T	15
			r1 = 6.3<br>r2 = 17.5	3.0 T	
	9	Gd-EOB-DTPA	r1 = 6.9<br>r2 = 8.7	1.5 T	15
			r1 = 6.2<br>r2 = 11.0	3.0 T	16
	10	Gd-BT-DO3A	r1 = 5.2<br>r2 = 6.8	1.5 T	15
			r1 = 5.0<br>r2 = 7.1	3.0 T	
	11	Gd-DTPA-BMA	r1 = 4.3<br>r2 = 5.2	1.5 T	15
			r1 = 4.0<br>r2 = 5.6	3.0 T	
	12	Gd-HPDO3A	r1 = 4.1<br>r2 = 5.0	1.5 T	15
			r1 = 3.7<br>r2 = 5.7	3.0 T	

Cat.	No.	Material	Relaxivity (mM^{-1} s^{-1})	Field	Refs
	13	Gd-DOTA	r1 = 3.6<br>r2 = 4.3	1.5 T	15
			r1 = 3.5<br>r2 = 4.9	3.0 T	
	14	Gd-DTPA	r1 = 3.6<br>r2 = 4.3	1.5 T	17
			r1 = 3.5<br>r2 = 4 9	3.0 T	
	15	SPIO	r1 = 19.0<br>r2 = 64.9	1.5 T	18
			r1 = 9.5<br>r2 = 65.2	3.0 T	
Nanoparticles	16	Mn@CCs	r1 = 42.9	1.5 T	19
			r1 = 31.1	7.0 T	
			r1 = 22.1	9.4 T	
	17	Mn-GA@BSA@DA	r1 = 18.5<br>r2 = 50.5	3.0 T	20
	18	Mn-CBP20	r1 = 16.3<br>r2 = 39.3	1.41 T	21
			r1 = 9.23	3.0 T	
			r1 = 5.69	4.7 T	
	19	H-MnO_2-PEG/C&D	r1 = 8.74	3.0 T	22
	20	PB: Mn nanocubes	r1 = 7.64	3.0 T	23
	21	HoMn-Ce6 NPs	r2 = 12.38	1.5 T	24
			r1 = 5.45<br>r2 = 14.92	3.0 T	
			r2 = 90.83	9.4 T	
	22	Fe_3O_4/MnO–Cy5.5–CTX	r1 = 5.37<br>r2 = 203.82	7.0 T	25
	23	PFC-73-Mn	r1 = 5.03	3.0 T	26
	24	DMM	r1 = 4.98	3.0 T	27
	25	Fe0.6 Mn0.4 O	r1 = 4.9<br>r2 = 61.2	7.0 T	28
	26	MnO-TETT-FA	r1 = 4.83	7.0 T	29
	27	PEGMnCaP	r1 = 4.73	1.0 T	30
	28	SPIO@SiO_2@MnO_2	r1 = 4.60<br>r2 = 82.21	3.0 T	31
	29	MnWOX-PEG	r1 = 3.37	3.0 T	32
	30	APPAM@U-104	r1 = 2.67<br>r2 = 16.2	7.0 T	33
	31	MnO and DTXco-loaded PTNPs	r1 = 2.40	7.0 T	34
	32	HMON	r1 = 1.42<br>r2 = 7.74	3.0 T	35

Cat.	No.	Material	Relaxivity (mM^{-1} s^{-1})	Field	Refs
	33	Fe_3O_4@MnO/mSiO_2-CD133	r1 = 0.99<br>r2 = 143.54	4.7 T	36
	34	RMn-NBs	r1 = 0.54<br>r2 = 11.25	7.0 T	37
Coordination complexes	35	MnLDPA-Zn_2	r1 = 12.5	1.5 T	38
			r1 = 11.54	9.4 T	
	36	Mn-JED	r1 = 8.9	1.41 T	39
			r1 = 4.8	4.7 T	
			r1 = 5.0	11.7 T	
	37	Mn-FBP	r1 = 8.5	1.4 T	40
	38	Mn-DPAC12A	r1 = 8.5	0.47 T	41
	39	Mn-H_4qtp_2-Br_2	r1 = 7.17	3.0 T	42
	40	Mn-PC2A-BP	r1 = 4.96	0.49 T	43
	41	Mn-PC2A-EA	r1 = 4.16<br>r2 = 6.12	0.49 T	44
			r1 = 3.26<br>r2 = 9.09	1.41 T	
			r1 = 4.14<br>r2 = 8.34	3.0 T	
	42	Mn-PhDTA	r1 = 3.72	0.5 T	45
	43	Mn-NOTA-NP	r1 = 3.57<br>r2 = 18.08	3.0 T	46
	44	Mn-DPAAA	r1 = 3.5	11.7 T	41
	45	Mn-EDTA-BTA	r1 = 3.5<br>r2 = 4.9	1.5 T	47
	46	Mn-OPMMA	r1 = 3.48<br>r2 = 5.85	0.41 T	47
	47	Mn-CyHBET	r1 = 3.3<br>r2 = 6.0	1.4 T	48
	48	Mn-3,9-PC2A	r1 = 2.91<br>r2 = 3.96	0.49 T	49
			r1 = 3.26<br>r2 = 6.34	1.41 T	
	49	Mn-HBET	r1 = 3.1<br>r2 = 11.1	1.4 T	48
	50	Mn-PC2A-EOB	r1 = 2.83<br>r2 = 5.87	1.41 T	50
	51	Mn-3,6-PC2A	r1 = 2.72<br>r2 = 3.49	0.49 T	49
			r1 = 2.98<br>r2 = 4.95	1.41 T	
	52	Mn-EDTA-EOB	r1 = 2.3<br>r2 = 4.3	1.5 T	17
	53	Mn-PyC3A	r1 = 2.1	1.4 T	40

References:

(1) Kühne, T. D.; Iannuzzi, M.; Ben, M. D.; Rybkin, V. V.; Seewald, P.; Stein, F.; Laino, T.; Khaliullin, R. Z.; Schütt, O.; Schiffmann, F.; et al. "CP2K: An electronic structure and molecular dynamics software package - Quickstep: Efficient and accurate electronic structure calculations," *The Journal of Chemical Physics* **2020**, *152* (19), 194103. DOI: 10.1063/5.0007045.

(2) Perdew, J. P.; Burke, K.; Ernzerhof, M. "Generalized gradient approximation made simple," *Physical Review Letters* **1996**, *77* (18), 3865-3868. DOI: 10.1103/PhysRevLett.77.3865.

(3) Grimme, S.; Ehrlich, S.; Goerigk, L. "Effect of the damping function in dispersion corrected density functional theory," *Journal of Computational Chemistry* **2011**, *32* (7), 1456-1465. DOI: 10.1002/jcc.21759.

(4) Grimme, S. "Density functional theory with London dispersion corrections," *WIREs Computational Molecular Science* **2011**, *1* (2), 211-228. DOI: 10.1002/wcms.30.

(5) Goedecker, S.; Teter, M.; Hutter, J. "Separable dual-space Gaussian pseudopotentials," *Physical Review B* **1996**, *54* (3), 1703-1710. DOI: 10.1103/PhysRevB.54.1703.

(6) VandeVondele, J.; Hutter, J. Gaussian basis sets for accurate calculations on molecular systems in gas and condensed phases. *The Journal of Chemical Physics* **2007**, *127* (11), 114105. DOI: 10.1063/1.2770708.

(7) Lu, T.; Chen, F. "Multiwfn: A multifunctional wavefunction analyzer," *Journal of Computational Chemistry* **2012**, *33* (5), 580-592. DOI: 10.1002/jcc.22885.

(8) Lu, T. "A comprehensive electron wavefunction analysis toolbox for chemists, Multiwfn.," *The Journal of Chemical Physics* **2024**, *161* (8). DOI: 10.1063/5.0216272 .

(9) Luo, Q.; Liu, J.; Ma, Q.; Xu, S.; Wang, L. "Single-atom Gd nanoprobes for self-confirmative MRI with robust stability," *Small* **2023**, *19* (23), 2206821.DOI:10.1002/smll.202206821

(10) Luo, Q.; Ma, Q.; Liu, T.; Luo, Y.; Wang, L.; Guo, C.; Wang, L. "Improving magnetic resonance imaging and chemodynamic therapy properties via tuning the Fe (II)/Fe (III) ratio in hydrophilic single-atom nanobowls," *ACS Nano* **2024**, *18* (14), 10063-10073. DOI: 10.1021/acsnano.3c12305

(11) Liu, S.; Jiang, Y.; Liu, P.; Yi, Y.; Hou, D.; Li, Y.; Liang, X.; Wang, Y.; Li, Z.; He, J.; Rong H.; Wang D.; Zhang J. "Single-atom gadolinium nano-contrast agents with high stability for tumor

T1 magnetic resonance imaging," *ACS Nano* **2023**, *17* (9), 8053-8063. DOI: 10.1021/acsnano.2c09664.

(12) Gao, F.; Fu, Q.; Ruan, Y.; Li, C.; Wang, Y.; Li, H.; Li, J.; Jiang, Y. "Elucidating manganese single-atom doping: strategies for fluorescence enhancement in water-soluble red-emitting carbon dots and applications for FL/MR dual mode imaging," *Advanced Science* **2025**, *12* (8), 2414895. DOI: 10.1002/advs.202414895.

(13) Elizondo, G.; Fretz, C. J.; Stark, D. D.; Rocklage, S. M.; Quay, S. C.; Worah, D.; Tsang, Y. M.; Chen, M. C.; Ferrucci, J. T. "Preclinical evaluation of MnDPDP: new paramagnetic hepatobiliary contrast agent for MR imaging," Radiology 1991, 178 (1), 73-78. DOI:10.1148/radiology.178.1.1898538

(14) Martin, M. N.; Jordanova, K. V.; Kos, A. B.; Russek, S. E.; Keenan, K. E.; Stupic, K. F. "Relaxation measurements of an MRI system phantom at low magnetic field strengths," *Magnetic Resonance Materials in Physics, Biology and Medicine* **2023**, *36* (3), 477-485. DOI: 10.1007/s10334-023-01086-y.

(15) Rohrer, M.; Bauer, H.; Mintorovitch, J.; Requardt, M.; Weinmann, H.-J. "Comparison of magnetic properties of MRI contrast media solutions at different magnetic field strengths," *Investigative Radiology* **2005**, *40* (11), 715-724. DOI:10.1097/01.rli.0000184756.66360.d3

(16) Pintaske, J.; Martirosian, P.; Graf, H.; Erb, G.; Lodemann, K.-P.; Claussen, C. D.; Schick, F. "Relaxivity of gadopentetate dimeglumine (Magnevist), gadobutrol (Gadovist), and gadobenate dimeglumine (MultiHance) in human blood plasma at 0.2, 1.5, and 3 Tesla," *Investigative Radiology* **2006**, *41* (3), 213-221. DOI:10.1097/01.rli.0000197668.44926.f7

(17) Islam, M. K.; Kim, S.; Kim, H. K.; Kim, Y. H.; Lee, Y. M.; Choi, G.; Baek, A. R.; Sung, B. K.; Kim, M.; Cho, A. E.; Kang H.; Lee G.; Choi S. H.; Lee T.; Park J. A.; Chang Y. "Synthesis and evaluation of manganese(ii)-based ethylenediaminetetraacetic acid-ethoxybenzyl conjugate as a highly stable hepatobiliary magnetic resonance imaging contrast agent," *Bioconjug Chem* **2018**, *29* (11), 3614-3625. DOI: 10.1021/acs.bioconjchem.8b00560.

(18) Knobloch, G.; Colgan, T.; Wiens, C. N.; Wang, X.; Schubert, T.; Hernando, D.; Sharma, S. D.; Reeder, S. B. "Relaxivity of ferumoxytol at 1.5 T and 3.0 T," *Investigative Radiology* **2018**, *53* (5), 257-263. DOI: 10.1097/RLI.0000000000000434.

(19) Qin, R.; Li, S.; Qiu, Y.; Feng, Y.; Liu, Y.; Ding, D.; Xu, L.; Ma, X.; Sun, W.; Chen, H. "Carbonized paramagnetic complexes of Mn (II) as contrast agents for precise magnetic resonance imaging of sub-millimeter-sized orthotopic tumors," *Nature Communications* **2022**, *13* (1), 1938. DOI:10.1038/s41467-022-29586-w

(20) Wu, C.; Zhong, J.; Li, J.; Luo, Y.; Wang, J.; Zeng, X.; Mao, J.; Lu, J.; Xu, J.; Wu, C.; et al. "Facile construction of manganese-based contrast agent with high T1 relaxivity for magnetic resonance imaging via flash technology-based self-assembly," *Regen Biomater* **2025**, *12*, rbaf009. DOI: 10.1093/rb/rbaf009.

(21) Zhang, C.; Ma, H.; DeRoche, D.; Gale, E. M.; Pantazopoulos, P.; Rotile, N. J.; Diyabalanage, H.; Humblet, V.; Caravan, P.; Zhou, I. Y. "Manganese-based type I collagen-targeting MRI probe for in vivo imaging of liver fibrosis," *npj Imaging* **2025**, *3* (1), 14. DOI: 10.1038/s44303-025-00075-1.

(22) Yang, G.; Xu, L.; Chao, Y.; Xu, J.; Sun, X.; Wu, Y.; Peng, R.; Liu, Z. "Hollow MnO_2 as a tumor-microenvironment-responsive biodegradable nano-platform for combination therapy favoring antitumor immune responses," *Nature Communications* **2017**, *8* (1). DOI: 10.1038/s41467-017-01050-0.

(23) Zhu, W.; Liu, K.; Sun, X.; Wang, X.; Li, Y.; Cheng, L.; Liu, Z. "Mn^{2+}-doped prussian blue nanocubes for bimodal imaging and photothermal therapy with enhanced performance," *ACS Applied Materials & Interfaces* **2015**, *7* (21), 11575-11582. DOI: 10.1021/acsami.5b02510.

(24) Cheng, Q.; Chang, Y.; Zhang, D.; Zhao, X.; Xiao, Z.; Chen, T.; Shi, C.; Luo, L. "Biomineralization synthesis of HoMn nanoparticles for ultrahigh-field-tailored and T1-T2 dual-mode MRI-guided cancer theranostics," *ACS Nano* **2024**, *18* (41), 27853-27868. DOI: 10.1021/acsnano.4c00516.

(25) Li, S.; Shao, C.; Gu, W.; Wang, R.; Zhang, J.; Lai, J.; Li, H.; Ye, L. "Targeted imaging of brain gliomas using multifunctional Fe_3O_4/MnO nanoparticles," *RSC Advances* **2015**, *5* (42), 33639-33645, 10.1039/C5RA01069A. DOI: 10.1039/C5RA01069A.

(26) Ouyang, S.; Chen, C.; Lin, P.; Wu, W.; Chen, G.; Li, P.; Sun, M.; Chen, H.; Zheng, Z.; You, Y.; Lv S.; Zhao P.; Lin B.; Tao J. "Hydrogen-Bonded organic frameworks chelated manganese for precise magnetic resonance imaging diagnosis of cancers," *Nano Letters* **2023**, *23* (18), 8628-8636. DOI: 10.1021/acs.nanolett.3c02466.

(27) Li, D.; Chen, J.; Lu, Y.; Yan, X.; Yang, X.; Zhang, F.; Tang, Y.; Cao, M.; Wang, J.; Pan, M.; Su C.; Shen J. "Codelivery of dual gases with metal-organic supramolecular cage-based microenvironment-responsive nanomedicine for atherosclerosis therapy," *Small* **2024**, *20* (40), e2402673. DOI: 10.1002/smll.202402673.

(28) Liu, X. L.; Ng, C. T.; Chandrasekharan, P.; Yang, H. T.; Zhao, L. Y.; Peng, E.; Lv, Y. B.; Xiao, W.; Fang, J.; Yi, J. B.; Zhang H.; Chuang K. H.; Bay B. H.; Ding J.; Fan H. M. "Synthesis of ferromagnetic $Fe_{0.6}$ $Mn_{0.4}$ o nanoflowers as a new class of magnetic theranostic platform for in vivo T1-T2 dual-mode magnetic resonance imaging and magnetic hyperthermia therapy," *Advanced Healthcare Materials* **2016**, *5* (16), 2092-2104. DOI: 10.1002/adhm.201600357.

(29) Chen, N.; Shao, C.; Qu, Y.; Li, S.; Gu, W.; Zheng, T.; Ye, L.; Yu, C. "Folic acid-conjugated MnO nanoparticles as a T1 contrast agent for magnetic resonance imaging of tiny brain gliomas," *ACS Applied Materials & Interfaces* **2014**, *6* (22), 19850-19857. DOI: 10.1021/am505223t.

(30) Mi, P.; Kokuryo, D.; Cabral, H.; Wu, H.; Terada, Y.; Saga, T.; Aoki, I.; Nishiyama, N.; Kataoka, K. "A pH-activatable nanoparticle with signal-amplification capabilities for non-invasive imaging of tumour malignancy," *Nature Nanotechnology* **2016**, *11* (8), 724-730. DOI: 10.1038/nnano.2016.72.

(31) Lu, H.; Chen, A.; Zhang, X.; Wei, Z.; Cao, R.; Zhu, Y.; Lu, J.; Wang, Z.; Tian, L. "A pH-responsive T1-T2 dual-modal MRI contrast agent for cancer imaging," *Nature Communications* **2022**. DOI: 10.1038/s41467-022-35655-x.

(32) Gong, F.; Cheng, L.; Yang, N.; Betzer, O.; Feng, L.; Zhou, Q.; Li, Y.; Chen, R.; Popovtzer, R.; Liu, Z. "Ultrasmall oxygen-deficient bimetallic oxide mnwox nanoparticles for depletion of endogenous GSH and enhanced sonodynamic cancer therapy," *Advanced Materials* **2019**, *31* (23), e1900730. DOI: 10.1002/adma.201900730.

(33) Fan, K.; Yang, X.; Tian, F.-z.; Li, S.-y.; Xu, T.-t.; Zhang, J.-q.; Xie, J.-b.; Ju, S.-h. "Acidic tumor microenvironment-activated MRI nanoprobes for modulation and visualization of anti-PD-L1 immunotherapy," *Nano Today* **2024**, *54*, 102146. DOI: https://doi.org/10.1016/j.nantod.2023.102146.

(34) Abbasi, A. Z.; Prasad, P.; Cai, P.; He, C.; Foltz, W. D.; Amini, M. A.; Gordijo, C. R.; Rauth, A. M.; Wu, X. Y. "Manganese oxide and docetaxel co-loaded fluorescent polymer nanoparticles for dual modal imaging and chemotherapy of breast cancer," *Journal of Controlled Release* **2015**, *209*, 186-196. DOI: 10.1016/j.jconrel.2015.04.020.

(35) Shin, J.; Anisur, R. M.; Ko, M. K.; Im, G. H.; Lee, J. H.; Lee, I. S. "Hollow manganese oxide nanoparticles as multifunctional agents for magnetic resonance imaging and drug delivery," *Angewandte Chemie International Edition* **2009**, *48* (2), 321-324. DOI: 10.1002/anie.200802323.

(36) Peng, Y.-K.; Lui, C. N. P.; Chen, Y.-W.; Chou, S.-W.; Raine, E.; Chou, P.-T.; Yung, K. K. L.; Tsang, S. C. E. "Engineering of single magnetic particle carrier for living brain cell imaging: a tunable T1-/T2-/dual-modal contrast agent for magnetic resonance imaging application," *Chemistry of Materials* **2017**, *29* (10), 4411-4417. DOI: 10.1021/acs.chemmater.7b00884.

(37) Huang, Z.; Huang, S.; Song, S.; Ding, Y.; Zhou, H.; Zhang, S.; Weng, L.; Zhang, Y.; Hu, Y.; Yuan, A.; et al. "Two-dimensional coordination risedronate-manganese nanobelts as adjuvant for cancer radiotherapy and immunotherapy," *Nature Communications* **2024**, *15* (1), 8692. DOI: 10.1038/s41467-024-53084-w.

(38) Anbu, S.; Kenning, L.; Stasiuk, G. J. "ATP-responsive Mn(II)-based T1 contrast agent for MRI," *Chemical Communications* **2023**, *59* (91), 13623-13626. DOI: 10.1039/d3cc03430e.

(39) Gale, E. M.; Jones, C. M.; Ramsay, I.; Farrar, C. T.; Caravan, P. "A janus chelator enables biochemically responsive MRI contrast with exceptional dynamic range," *Journal of the American Chemical Society* **2016**, *138* (49), 15861-15864. DOI: 10.1021/jacs.6b10898.

(40) Gale, E. M.; Atanasova, I. P.; Blasi, F.; Ay, I.; Caravan, P. "A manganese alternative to gadolinium for MRI Contrast," *Journal of the American Chemical Society* **2015**, *137* (49), 15548-15557. DOI: 10.1021/jacs.5b10748.

(41) Forgács, A.; Pujales-Paradela, R.; Regueiro-Figueroa, M.; Valencia, L.; Esteban-Gómez, D.; Botta, M.; Platas-Iglesias, C. "Developing the family of picolinate ligands for Mn^{2+} complexation," *Dalton Transactions* **2017**, *46* (5), 1546-1558. DOI: 10.1039/C6DT04442E.

(42) Yu, M.; Ward, M. B.; Franke, A.; Ambrose, S. L.; Whaley, Z. L.; Bradford, T. M.; Gorden, J. D.; Beyers, R. J.; Cattley, R. C.; Ivanović-Burmazović, I.; Schwartz D. D.; Goldsmith C. R. "Adding a second quinol to a redox-responsive MRI contrast agent improves its relaxivity response to H_2O_2." *Inorganic Chemistry* **2017**, *56* (5), 2812-2826. DOI: 10.1021/acs.inorgchem.6b02964.

(43) Kálmán, F. K.; Nagy, V.; Váradi, B.; Garda, Z.; Molnár, E.; Trencsényi, G.; Kiss, J.; Même, S.; Même, W.; Tóth, É.; et al. "Mn(II)-based MRI contrast agent candidate for vascular imaging," *Journal of Medicinal Chemistry* **2020**, *63* (11), 6057-6065. DOI: 10.1021/acs.jmedchem.0c00197.

(44) Botár, R.; Molnár, E.; Trencsényi, G.; Kiss, J.; Kálmán, F. K.; Tircsó, G. "Stable and inert Mn(II)-based and pH-responsive contrast agents," *Journal of the American Chemical Society* **2020**, *142* (4), 1662-1666. DOI: 10.1021/jacs.9b09407.

(45) Pota, K.; Garda, Z.; Kálmán, F. K.; Barriada, J. L.; Esteban-Gómez, D.; Platas-Iglesias, C.; Tóth, I.; Brücher, E.; Tircsó, G. "Taking the next step toward inert Mn^{2+} complexes of open-chain ligands: the case of the rigid PhDTA ligand," *New Journal of Chemistry* **2018**, *42* (10), 8001-8011, 10.1039/C8NJ00121A. DOI: 10.1039/C8NJ00121A.

(46) Islam, M. K.; Baek, A. R.; Yang, B. W.; Kim, S.; Hwang, D. W.; Nam, S. W.; Lee, G. H.; Chang, Y. "Manganese (II) complex of 1,4,7-triazacyclononane-1,4,7-triacetic acid (NOTA) as a hepatobiliary MRI contrast agent," *Pharmaceuticals (Basel)* **2023**, *16* (4). DOI: 10.3390/ph16040602.

(47) Debretsion, A. E.; Bunda, S.; Lihi, N.; Garda, Z.; Van Doorslaer, S.; Kun, E.; Csupász, T.; Tircsó, G.; Tóth, É.; Kálmán, F. K. "Stability and relaxometric characterization of a manganase(II) based macrocyclic complex containing malonate pendant," *Dalton Transactions* **2025**, *54* (27), 10751-10760. DOI: 10.1039/d5dt01056j.

(48) Gale, E. M.; Mukherjee, S.; Liu, C.; Loving, G. S.; Caravan, P. "Structure-redox-relaxivity relationships for redox responsive manganese-based magnetic resonance imaging probes," *Inorganic Chemistry* **2014**, *53* (19), 10748-10761. DOI: 10.1021/ic502005u.

(49) Garda, Z.; Molnár, E.; Hamon, N.; Barriada, J. L.; Esteban-Gómez, D.; Váradi, B.; Nagy, V.; Pota, K.; Kálmán, F. K.; Tóth, I.; Lihi N.; Platas-Iglesias C.; Tóth É.; Tripier R.; Tircsó G. "Complexation of Mn(II) by rigid pyclen diacetates: equilibrium, kinetic, relaxometric, density functional theory, and superoxide dismutase activity studies," *Inorg Chem* **2021**, *60* (2), 1133-1148. DOI: 10.1021/acs.inorgchem.0c03276.

(50) Hall, R. C.; Qin, J.; Laney, V.; Ayat, N.; Lu, Z.-R. "Manganese(II) EOB-pyclen diacetate for liver-specific MRI," *ACS Applied Bio Materials* **2022**, *5* (2), 451-458. DOI: 10.1021/acsabm.1c01259.
